# Neurodevelopment Genes in Lampreys Reveal Trends for Forebrain Evolution in Craniates

**DOI:** 10.1371/journal.pone.0005374

**Published:** 2009-04-28

**Authors:** Adèle Guérin, Yves d'Aubenton-Carafa, Emna Marrakchi, Corinne Da Silva, Patrick Wincker, Sylvie Mazan, Sylvie Rétaux

**Affiliations:** 1 Equipe Développement Evolution du Cerveau Antérieur, UPR2197-CNRS, Institut Alfred Fessard, Gif sur Yvette, France; 2 Centre de Génétique Moléculaire (CNRS), Gif-sur-Yvette, France; 3 Génoscope (CEA), UMR8030, CNRS and Université d'Evry, Evry, France; 4 Développement et Evolution des vertébrés, UMR 6218, CNRS and Université d'Orléans, Orléans, France; University of Maryland, United States of America

## Abstract

The forebrain is the brain region which has undergone the most dramatic changes through vertebrate evolution. Analyses conducted in lampreys are essential to gain insight into the broad ancestral characteristics of the forebrain at the dawn of vertebrates, and to understand the molecular basis for the diversifications that have taken place in cyclostomes and gnathostomes following their splitting. Here, we report the embryonic expression patterns of 43 lamprey genes, coding for transcription factors or signaling molecules known to be involved in cell proliferation, stemcellness, neurogenesis, patterning and regionalization in the developing forebrain. Systematic expression patterns comparisons with model organisms highlight conservations likely to reflect shared features present in the vertebrate ancestors. They also point to changes in signaling systems –pathways which control the growth and patterning of the neuroepithelium-, which may have been crucial in the evolution of forebrain anatomy at the origin of vertebrates.

## Introduction

Lampreys are a key species to study the evolution of morphological traits at the emergence of craniates (or vertebrates). As agnathans (meaning without jaws) and cyclostomes (meaning round mouth), they are, together with hagfish whose embryology is less advanced [1], the only extant representatives of one of the two major taxa which arose in the vertebrate lineage about 500 million years ago and gave rise to the two sister groups of vertebrates: the agnathans and the gnathostomes. The description of shared characters between lampreys and the more traditional vertebrate model organisms -all belonging to gnathostomes- is therefore crucial to address the emergence of the novelties, which characterize vertebrates among chordates, and the amazing diversification which arose within the taxon [2].

Lampreys are odd animals, with a peculiar anatomy, a remarkably long and special life cycle, and an extended embryonic and larval development when compared to the other widely-used “developmental biology models” (reviewed in [2], [3]). In particular, the brain of lampreys shows a number of unique characteristics –for example it lacks myelin [4]-, although its overall aspect is clearly vertebrate-like. Indeed, and contrarily to other non-vertebrate chordates like tunicates and amphioxus, the lamprey brain includes a real telencephalon, a forebrain region that is derived from the anterior-most part of the alar plate of the neural tube and constitutes a vertebrate synapomorphy. Nevertheless, the lamprey telencephalon is significantly different from gnathostome-type telencephalon during development and in adults, in terms of size, shape, cytoarchitecture, and neuroanatomy. To begin with, the embryonic lamprey telencephalon is remarkably tiny (in relative proportions versus other brain regions) and shows very slow and weak growth, and the resulting adult telencephalon is strikingly cell poor. Second, the lamprey telencephalon (like the rest of its brain) is not migrated, meaning that the neuronal cell bodies remain in a ventricular position and do not invade the brain tissue after they are born. This results in a very basic cytoarchitecture, with nearly all neurons packed in periventricular position [5], a feature which strongly contrasts with the extensive migrations in both radial and tangential direction which take place in gnathostome telencephalon, generating complex cytoarchitectonic laminar or nuclear arrangements [6], [7], [8], [9]. Third, the lamprey telencephalon is only partially evaginated and a large part of it is called the “telencephalon impar” [5] because it does not inflate through the same morphogenetic movements as its lateral-most part. The non-evaginated telencephalon is therefore similar in terms of morphogenesis to the diencephalon. Fourth, the lamprey subpallium does not present one of the two major subpallial components described in gnathostomes, and lacks a pallidum [10]. We and others have discussed the absence of a pallidum in adults with respect to the absence of expression of positional and regional identity factors [11], [12] and signaling molecules [12] during early embryonic development.

Here, we report an exhaustive molecular characterization of the lamprey embryonic forebrain, aimed at understanding the molecular and cellular mechanisms that shape this territory. Expression analyses of more than 40 genes known to control proliferation, neurogenesis and patterning in the forebrain of gnathostomes, were conducted in lamprey embryos, starting from pre-hatching to larval stages. Systematic comparisons with vertebrate model organisms suggest that changes in signaling systems –pathways which control the growth and patterning of the neuroepithelium- have been crucial in the evolution of forebrain anatomy at the dawn of vertebrates.

## Results and Discussion

In order to identify lamprey genes involved in forebrain development, we used a candidate approach focused on genes known to be involved in the control of cell proliferation, neurogenesis, regionalisation of the central nervous development, including components of the major signaling pathways. Searches in our cDNA database using zebrafish sequences as queries led to the identification of 89 specific hits. Following further identification by reverse Blast and phylogenetic analyses, these hits could be assigned to a total of 43 distinct genes described in Table 1.

**Table 1:** List of the 89 lamprey clones whose phylogeny and expression was studied in the present paper.

Clone #	Orthology	Species	Clone #	Orthology	Species
***Proliferation/stem cell (20clones)***			***Regionalization (continued…)***		
31,131,132,140	PCNA	*P.m.*	17	FoxB1	*L.f*
40	Musashi2	*L.f.*	130	FoxC1/2	
168, 169	Pisolo	*L.f. and P.m*	37	SoxE2 (Sox 8/9/10)	*L.f*
49	Notch	*P.m.*	115, 117, 120	SoxE3 (Sox8)	*L.f*
48, 133	Delta1	*P.m. and L.f*	116	SoxD (Sox 5/6/13)	*L.f*
152–154	Cyp17	*L.f.*	122	SoxD1 (Sox 5/6/13)	*L.f*
38, 42	SoxB1 (1/2/3)	*L.f.*	41	Sox ?	*L.f*
28, 137	Sox3	*L.f.*	24	Tcf7-like	*L.f*
146, 147	HMGbox	*L.f.*	***Midline/signaling (32 clones)***		
148	HMGbox	*L.f.*	161, 162, 163, 164	Pleiotrophin	*L.f. and P.m*
***Neurogenesis (6 clones)***			15,16,84,86,83,90,135	FgfR	*L.f. and P.m*
88	Neurogenin1	*L.f.*	gift Kate Hammond	Fgf8/17	*L.f*
18	NeuroD2	*L.f.*	13	Fgf ?	*L.f*
29, 123, 125, 127	Id2/3	*L.f.*	1, 2, 70	Wnt7	*L.f*
***Regionalisation/patterning (31 clones)***			73	Wnt5	*L.f*
22, 25	COUP-Tf/NR2F	*L.f.*	94, 101, 105, 107, 108	Frizzled1/2	*L.f. and P.m*
23, 93	OtxA-Otx1/2	*L.f.*	95, 99	Frizzled2/7	*L.f*
53	Otx5/Crx	*L.f.*	100, 136	Frizzled1/2/7	*L.f*
19, 64	Dbx1	*L.f. and P.m*	106	Frizzled5/8	*P.m*
114	LIM-kinase2	*L.f.*	102, 103	SFRP1/5	*L.f*
160	Ldb3	*P.m.*	96, 97, 98, 109	SFRP2	*L.f. and P.m*
8	Ldb1/CLIM1	*L.f.*			
113	Pitx2	*P.m.*			
71, 72, 74, 76–80	Pitx2	*L.f.*			
20	Six1/2	*L.f.*			
69	Pax3/7	*L.f.*	***Total clones***		***89***

Table 1 gives a list of clones whose expression was studied in the present paper. The family, the orthology relationship (when possible), and the species of origin (*P.m., Petromyzon marinus*; *L.f., Lampetra fluviatilis*) of the clones are given. Sequences are accessible in Genbank under accession numbers FP243278 to FP243259.

Whole-mount *in situ* hybridizations were carried out using each of the 89 probes on *Petromyzon marinus* embryos at stage (st.) 24 (hatching) and st. 26 (prolarvae), completed when necessary by slightly earlier or later stages (st. 19–22/tailbud or st. 27/ammocoete larvae). The distribution of the transcripts within the depth of the neuroepithelium as well as the presence of expression boundaries was further assessed on transverse and sagittal histological sections. The results obtained for genetic systems respectively involved in the control of cell proliferation, neurogenesis, regionalization and cell signaling are presented in the following sections, under a “results and discussion” format for the sake of simplicity and clarity.

### Proliferation and stem cells

**Figure 1:**
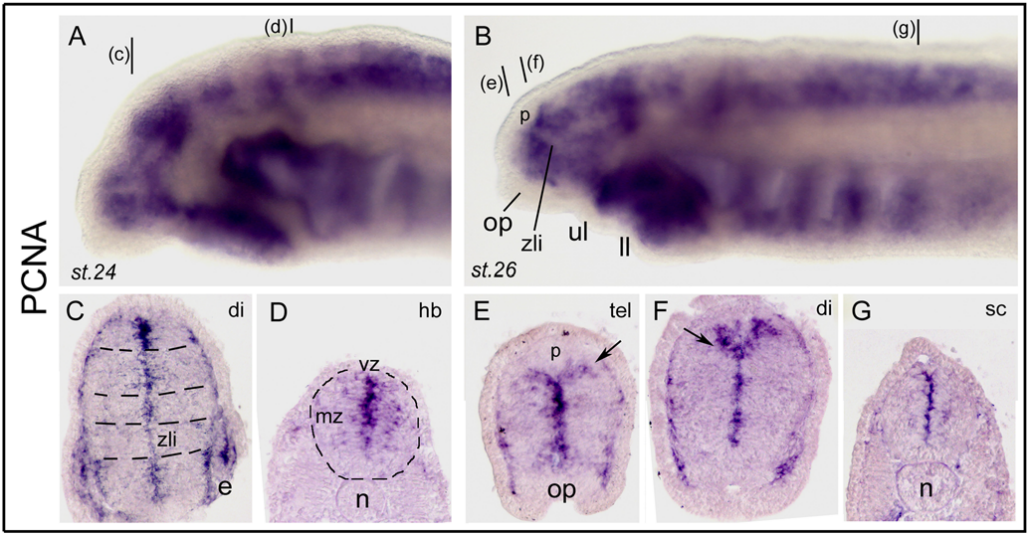
Developmental expression of lamprey PCNA. A and B show *in toto* views, and C to G show transverse sections. In this and subsequent figures, anterior is to the left and dorsal is up for *in toto* images, the stage is indicated in the bottom left corner (st.24, st.26, or else), and section level is indicated in the top right corner of the photography (e.g., tel for telencephalon, di for diencephalon; see code in Figure 2 inset). In this figure only and to help the reader through the paper, the planes of section are indicated by black bars in A for the sections in C and D, and by black bars in B for the sections shown in EFG. In C, the dotted lines highlight the transverse domains which emerge from PCNA expression. In D, the circle delineates the contours of the neural tube. In E and F, the arrows point to the typical fork-like pattern reproducibly found at the dorsal midline just posterior to the pineal gland (p) and which probably corresponds to the dorsal thalamus (see text). zli: *zona limitans intrathalamica*; mz, vz, marginal and ventricular zone. Pictures shown are from clones 31 and 132.

Proliferating Cell Nuclear Antigen (PCNA) labels cells in S phase of the cell cycle (plus G1/G2) and is probably the most widely used marker for proliferating/cycling cells throughout the organism, including the neural tube. PCNA immuno-histochemistry has been used to characterize proliferation patterns in the developing lamprey brain [13]. Here, we used in situ hybridization with four independent clones for the PCNA coding gene (all yielding identical results) to gain insight into proliferation patterns in the embryonic and larval forebrain. As shown in Figure 1, both st. 24 and st. 26 embryos showed a heavy expression as viewed *in toto*, with some “banded” aspects along the antero-posterior axis of the neural tube (Fig. 1A, B). As examined on sections, expression was very strong around the ventricular zone (vz, relatively thick), but much more diffuse in the mantle zone (mz), and showed some clear discontinuities separating vz progenitor domains (Fig. 1C, E, F, dotted lines on Fig. 1C). At rostral forebrain levels, a fork-like pattern of expression at the dorsal midline was a hallmark of the dorsal thalamus highly proliferative zone that lies behind the pineal organ (p in Fig. 1B, E) and the telencephalic/diencephalic boundary (Fig. 1E, F, arrow; see also below and dorsal midline markers). The discontinuous proliferation pattern observed along the neuraxis is particularly noteworthy, and prefigures the neurogenetic or histogenetic domains which themselves correspond to the future functional units of the larval and adult brain. In this context, proliferation is clearly absent in patterning centers such as the *zona limitans intrathalamica* (zli, Fig. 1B, C) which separates and patterns the prethalamic and thalamic regions of the diencephalon (see [12] for zli and *Hedgehog* in embryonic lampreys). Such an organization and pattern is highly similar to what has been reported in fish [14], [15], or using immunohistochemistry in lampreys [13]. Moreover, here we show that the restriction of proliferation to a small subset of ventricular cells is already well established at st.24. This finding, together with the estimated very long cell cycle length in lampreys [13] may partly explain the especially slow growth of its brain.

To be compared to PCNA, Musashi (a RNA-binding protein; reviewed in [16]), Notch, a single-pass transmembrane receptor for the ligand Delta, (reviewed in [17]), Pisolo (a newly discovered proliferation factor; Alessandro Alunni and Jean-Stéphane Joly, personal communication) and two Sox B1 subgroup members (HMG-box containing factor; reviewed in [18]) are markers of neural stem cells and neural progenitor cells which were present in our database (See also Figure S1 for high power pictures of expression data and Figure S2 for phylogeny).

**Figure 2:**
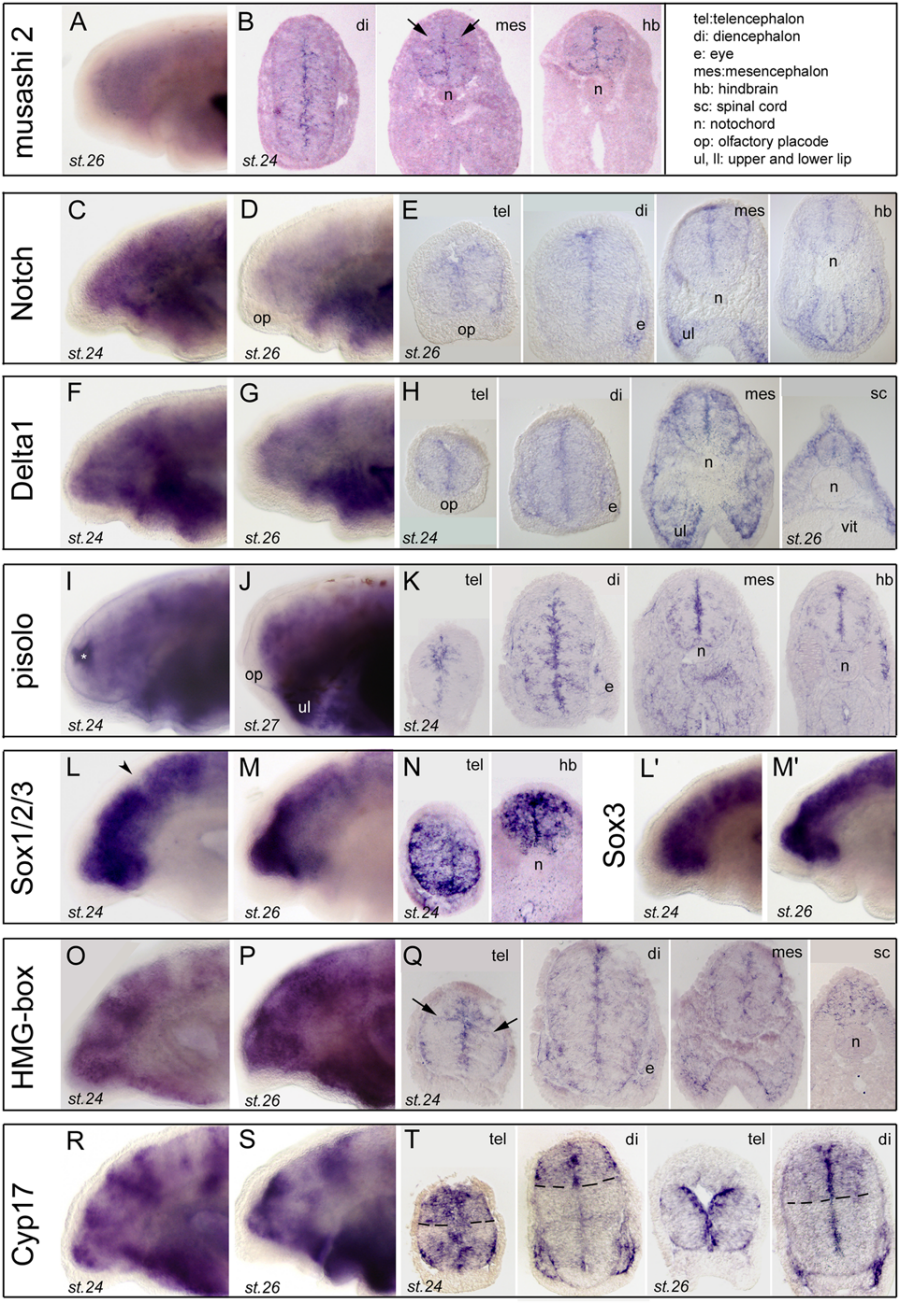
Expression of stem cell and proliferation markers in lamprey developing brain. A (toto) and B (sections) show lamprey musashi 2 (Msi2, clone 40). C,D (toto) and E (sections) show lamprey Notch (clone 49). F,G (toto) and H (sections) show lamprey Delta1 (clone 48). Vit, vitellus. I,J (toto) and K (sections) show lamprey pisolo (pictures taken from clone 169). The white asterisk in panel I indicates background trapping in the ventricle. L,M (toto) and N (sections) show a lamprey SoxB1 (Sox1/2/3) member, see also phylogeny for Sox genes in Figure S5). Toto views are from clone 38, and sections from clone 42. L',M' (toto) show another SoxB1 member identified as Sox3 (pictures taken from clone 137). O,P (toto) and Q (sections) show a lamprey HMG-box (toto view from clone 148, sections from clone 147). Arrows in panel 2Q indicate putative streams of radially migrating cells. R,S (toto) and T (sections) show lamprey Cyp17 (toto views are from clones 154 and 153, sections are from clone 152). Dotted lines in T highlight the “banded” pattern of expression of this transcript. The inset in the upper right corner gives the list of anatomical abbreviations used in this and other figures.

Musashi showed faint but clear expression along the ventricular lining (Fig. 2A, B), with a few expressing cells also consistently observed in the marginal portion of the neuroepithelium (often showing a bilateral symmetrical distribution, arrows on Fig. 2B). Notch mRNA expression was diffuse at st.24 and got nicely restricted to a “thick vz” pattern at st.26 (Fig. 2C–E). Its ligand Delta was similarly confined to the ventricular aspect of the neuroepithelium, with additional weak but significant expression in the marginal zone. Pisolo showed somewhat similar to musashi, salt and pepper-like expression (Fig. 2I–K), with robust signal in a “thick vz” but also many scattered expressing cells in the differentiating zone, which appeared quite randomly distributed, i.e., with absence of an “interpretable” pattern. Finally, two distinct Sox members (both belonging to the B1 group, see Figure S5, AB) showed strong and conspicuous signal throughout the depth of the neuroepithelium (vz to mz) at st.24 (Fig. 2L–N, L'–N'). Expression was then excluded from the marginal zone at st.26, remaining in an “enlarged vz” (interpreted as vz+svz) throughout the neural tube (not shown). These distributions are fully comparable to those of orthologous genes in fish for example (ZFIN database for zebrafish Msi, Notch, Delta, Sox2; Alunni and Joly for medaka pisolo). Of note, at the difference of PCNA, Musashi, Notch and Delta expression did not show obvious discontinuities in the A/P axis of the neural tube -although some patches and sub-domains may be observed for Musashi and Pisolo in the D/V axis at posterior levels. This is also consistent with data from fish embryos, in which no discontinuity along the neuraxis is described for these “stem cell” markers. By contrast, Sox expression displayed a clear A/P discontinuity at the mid-hindbrain boundary (mhb; e.g., arrowhead on Fig. 2L; also seen in zebrafish) and more posteriorly in the hindbrain and spinal cord. The fact that these typical neural stem cell markers are expressed in confined region of the neuroepithelium during embryogenesis in lampreys, and in a highly comparable manner when compared to studied gnathostome species, suggest that the molecular mechanisms controlling neural “stemcellness” and the renewal of neural progenitors are shared –at least in part- by all the craniates.

Our *in situ* hybridization screen also revealed striking and unexpected patterns for some genes not classically described as “proliferation genes”, but which may be classified as such due to their clear-cut patterns. Two of them are presented below.

These included two independent HMG-box family members (non Sox, poorly resolved phylogenetic relationship between the HMGb1 to 3 groups), which both showed an impressive and banded pattern *in toto* (Fig. 2O–Q), strongly resembling the PCNA pattern. Examination on sections confirmed the vz discontinuities (therefore similar to PCNA, compare to Fig. 1) in the A/P axis, and additionally revealed a complex pattern in the depth of the neuroepithelium (Fig. 2Q): both the ventricular and the marginal zone were positive for these two clones, leaving a non-expressing neuroepithelial zone at the level of the putative svz. Their pattern also showed some “trails” reminiscent of migration from the vz to the mz, i.e., in the radial dimension of the neuroepithelium (arrows on Fig. 2Q). This is interesting to relate to the comment of Villar-Cheda and colleagues [13], who suggested that there is little if any tangential migration in the lamprey brain. In line with this conclusion and although the occurrence of tangential migrations processes cannot be excluded at larval, metamorphic or even adult stages, our results suggest that radial migrations prevail at embryonic stages in the lamprey. Generally, tangential migrations are considered as a powerful means of generating diversity in the brain, they represent a shared mechanisms in the forebrain among gnathostomes including sharks [6], [9], [19], [20], and may therefore be a jawed vertebrate innovation.

In the same category, three independent clones for Cyp17 (the steroidogenic enzyme cytochrome P450 17alpha-hydroxylase) showed an A/P banded pattern with strong vz/lower svz-mz expression (Fig 2R–T). Clear expression boundaries and domains in the telencephalon (between pallial and subpallial areas, Fig. 2T) and diencephalon (between dorsal and ventral thalamic areas, Fig. 2T) were detected on sections, and expression became amplified and preferential in the forebrain at st.26. Together with Cyp19 (also called brain aromatase), Cyp17 belongs to the large superfamily of Cyp genes which have diverse functions in steroid, lipid, and xenobiotic metabolism [21], [22]. Cyp 17 and 19 are specifically involved in the synthesis of neurosteroids, and their expression is known to be particularly high in the brain of teleost fishes, in which the high production of neurosteroids has been related to the continuous neurogenesis through life [23]. They are also expressed during development in fish and amphibians, where their functions are more hypothetical [24]. The expression pattern found here for lamprey Cyp17 may suggest a putative important role in the control of proliferation and/or neurogenesis in specific forebrain domains as discussed in fish, and suggests that a significant synthesis of neurosteroids in the embryonic brain is a shared character in anamniote craniates.

Overall, the above described expression for proliferating progenitors/stem cells gene markers in the developing lamprey brain were globally highly similar to the situation described in jawed vertebrates (see summary Figure).

### Neurogenesis

**Figure 3:**
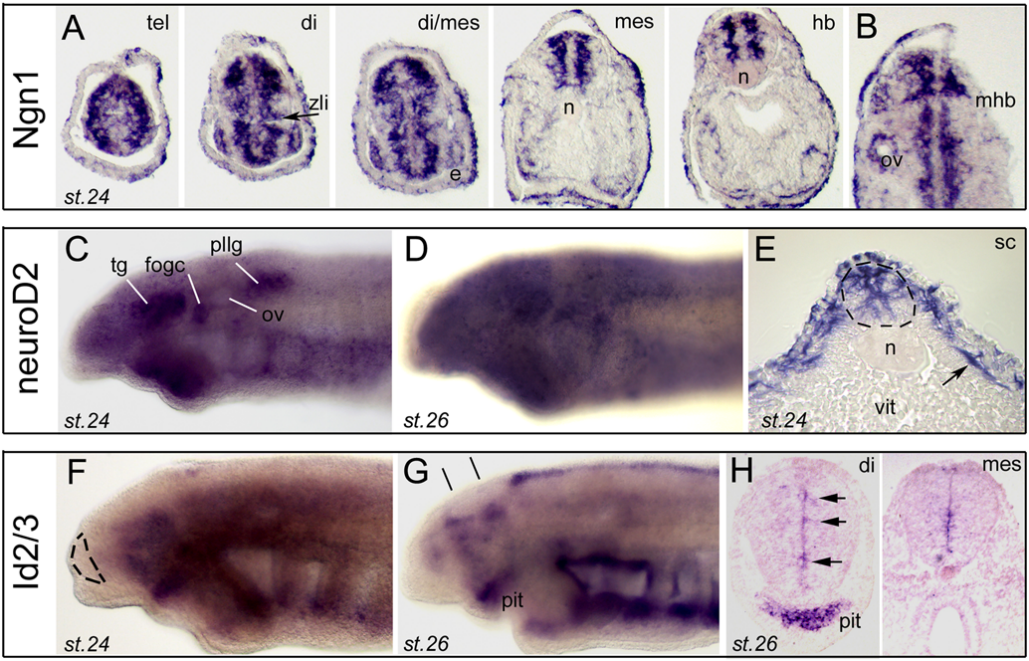
Developmental expression of proneural factors in lamprey brain. A and B show Ngn1 expression (clone 88). A shows a complete antero-posterior series through forebrain of a stage 24 embryo, and B shows a horizontal section. The zli (zona limitans intrathalamica) and mhb (mid-hindbrain boundary) signaling centers are indicated. ov, otic vesicle. C,D (toto) and E (section) show NeuroD2 expression (pictures from clone 18). The arrow in E points to NeuroD2-expressing post-migratory neural crest. vit, vitellus. tg, fog, and pllg point to the trigeminal, facial/octaval and posterior lateral line ganglia, respectively. See Barreiro-Iglesias et al. 2008 in the open Journal of Zoology (Open access, indexed in Google Scholar) for localization of the lamprey ganglia. ov, otic vesicle. F,G (toto) and H (sections) show Id2/3 expression (toto views from clone 123, sections from clone 127). The dotted lines in F delineate the telencephalon. The black lines in G indicate the section plane of sections in panel H. Arrows indicate concentration/patches of ventricular expression. pit, pituitary.

Our cDNA collection contained several independent clones for Neurogenin (Ngn1) and its downstream mediator NeuroD2 (see Figure S3 for phylogeny), which are two proneural basic helix-loop-helix factors and key regulators of vertebrate neurogenesis [25]. Expression of both transcription factors was, as expected for proneural factors, strikingly different from those of proliferation factors reported above (Fig. 3). Ngn1 showed strong expression throughout the neural tube, at the exclusion of the proliferative vz (Fig. 3A, compare to PCNA, Fig. 1C–G). A complete antero-posterior series shown in figure 3A demonstrates important discontinuities at mid-diencephalic level (putatively at the location of the zli organizer, arrowhead) and at the mid-hindbrain boundary (mhb), another important organizing center (Fig. 3B). NeuroD2 on the other hand displayed high expression throughout the nervous system, and only on lightly labeled embryos could be discerned a post-migratory neural crest–like pattern encompassing the condensing cranial ganglia (Fig. 3C–E). In the developing neural tube, NeuroD showed a well recognizable “neurogenic” and Ngn1-like pattern (Fig. 3E).

In this section we also report a lamprey Id2/3 (for Inhibitor of Differentiation) family member (Fig. 3F–H, see also next section on patterning). Id proteins are HLH factors without basic amino acid DNA binding domain, and act as dominant regulators of proneural genes, possibly intervening in the maintenance of neural stem cells [26], [27]. We found the lamprey Id2/3 transcripts in the ventricular zone and showing clear discontinuities, especially in the diencephalon (arrows in Fig. 3H). The patchy appearance of Id2/3 expression in lamprey (also discussed below), and its presence in the vz where stem cells and progenitors reside (Fig. 3H), are concordant with patterns reported in other species and in agreement with its suspected functional role. Of note, the present Id2/3 factor happens to be different from the Id gene previously reported by Meulemans et al. [28], suggesting that lampreys have at least two Id family members.

Thus, the molecular mechanisms for the genetic control of neurogenesis appear well conserved in craniates, with a clear ventricular to marginal progression of cells from proliferation to neuronal specification to neuronal differentiation, respectively. In addition, the absence of proliferation as well as neurogenesis at the level of zones suspected to be “signaling zones” or “secondary organizers” highlights the importance of these special centers to organize the surrounding neuroepithelium and control cell fate. It also suggests that the genetic networks to accomplish these crucial neuro-developmental processes were recruited in the craniate ancestors of lampreys and gnathostomes, because non-craniate chordates such as amphioxus or ascidians do not possess equivalent signaling centers in their anterior-most neural tube ([12] and see also below, section on “signaling”, and summary figure).

### Regionalisation and patterning

We and others have previously brought evidence for the (partial) conservation of the plan of organization of the developing brain in lampreys as compared to jawed vertebrates [11], [12], [29], [30], [31]. Our cDNA collection contained several clones of interest in this respect, which allow completing the picture of the patterning events during forebrain development in lampreys (see. Figure S4 for phylogenies).

**Figure 4:**
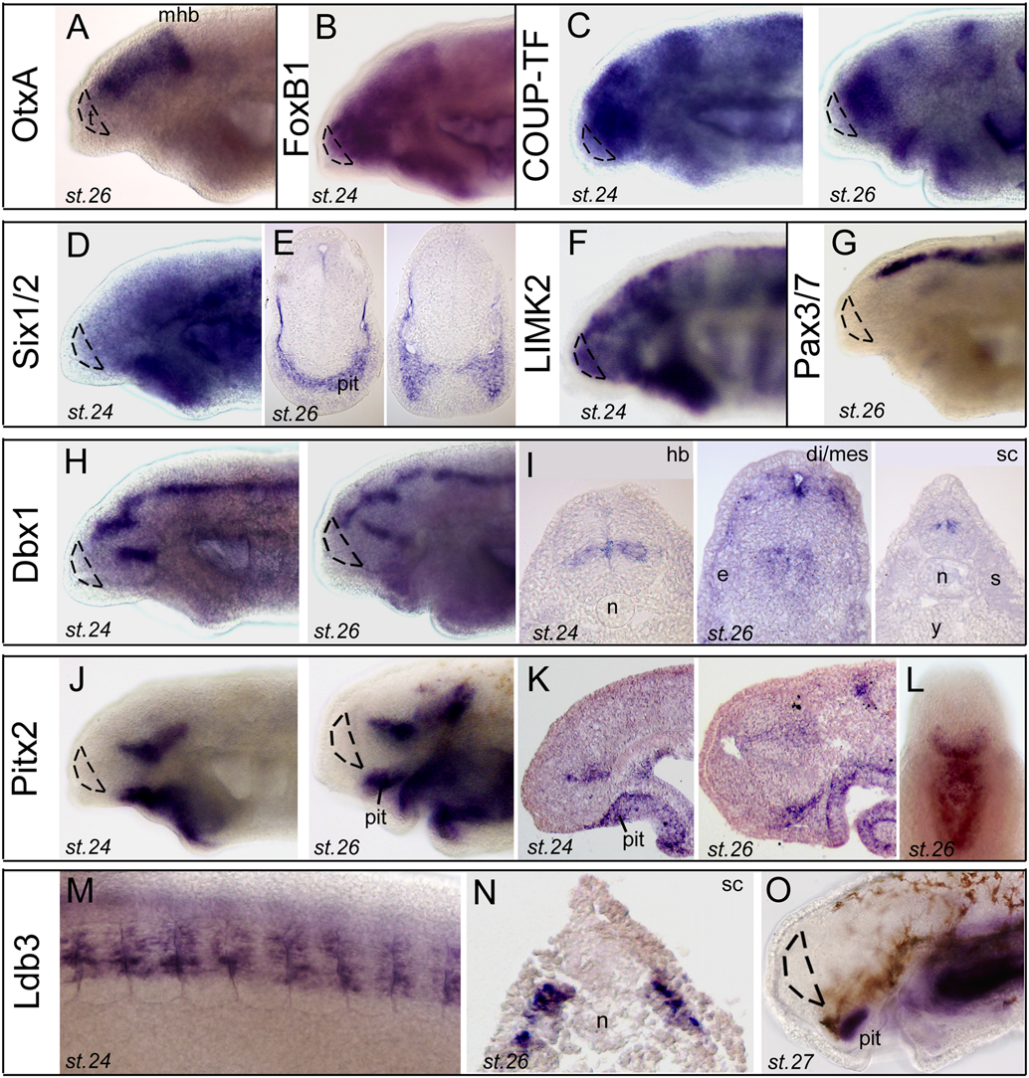
Developmental expression of regionalization and tissue patterning genes in lamprey. The dotted lines on *in toto* views delineate the telencephalon. pit, pituitary. A, OtxA (clone 93). The *Lampetra fluviatilis* clone shown is orthologous to the *Lampetra japonica* OtxA/Otx2 reported by Ueki et al. (1998) [33]. B, FoxB1 (clone 17). C, COUP-TF/NR2F (clone 25). D (toto) and E (sections) show Six1/2 expression (clone 20). Note the conspicuous expression in the eyes and pituitary (pit). F, LIMk2 (clone 114). G, Pax3/7 (clone 69). H (toto) and I (sections) show Dbx1 expression (clone 19). J (toto), K (sections) and L (toto, ventral view, anterior is up) show Pitx2 expression (clone 113). M,O (toto) and N (section) show Ldb3 expression (clone 160). M is a lateral view at trunk level. N is a transverse section to show expression in somites.

**Figure 5:**
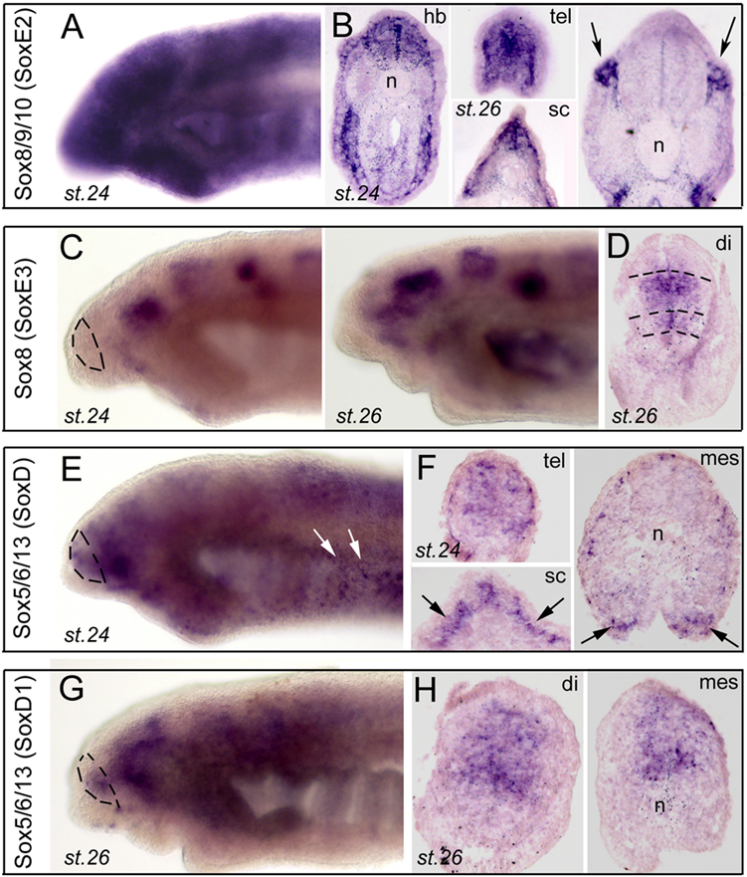
Developmental expression of four lamprey Sox family members. The dotted lines on *in toto* views delineate the telencephalon. A (toto) and B (sections) show a group E (Sox8/9/10) member (clone 37, orthologous to P.m SoxE2). Arrows point to neural crest-derived expressing ganglia. C (toto) and D (section) show Sox8 expression (clones 115,117,120, orthologous to L.j SoxE3). Shown are clones 115 (C, st.24) and 117 (C, st.26 and D). Dotted lines in D delineate the transverse diencephalic domains. E,G (toto) and F,H (sections) show expression of two lamprey group D (Sox5/6/13) members. Clone 116 (E,F) is expressed in both the brain and the neural crest (arrows in E and F), whereas clones 120 (G) and 122 (H) expression is restricted to the brain.

As described previously [32], [33], OtxA expression territory is reminiscent of the one of Otx1 and Otx2 in jawed vertebrates, spanning the forebrain and midbrain, with a sharp posterior boundary (Fig. 4A). Within the forebrain, a number of clones reported in Figure 4 highlighted transverse boundaries (i.e., in the A/P axis), consistent with a neuromeric/prosomeric mode of forebrain development in lampreys as well as in other vertebrates. Genes such as FoxB1 (Fig. 4B), COUP-TF/NR2F (Fig. 4C), Dbx1 (Fig. 4H), Pitx2 (Fig. 4JKL), LIM-kinase 2 (Fig. 4F) or Sox8/9 (Fig. 5C and see below) displayed banded patterns which confirmed the neuromeric nature of the embryonic brain (see also HMG-box and Cyp17 “banded” patterns on Fig. 2O, R). This was particularly striking in the diencephalon, where many of the genes shown in figures 4–5 displayed transverse and highly specific and nested expression domains. It is reasonable to suspect that these factors (among which a vast majority of transcription factors) confer regional identity and/or neuronal identity to expressing cells.

Remarkably, the telencephalon appeared to lack expression for several of this category of development regulators, although our search in the database was clearly oriented towards forebrain-specific genes. Indeed, neither COUP-TF (Fig. 4C), Dbx1 (Fig. 4G), Six1/2 (Fig. 4I) nor Id2/3 (Fig. 3F), were expressed in the lamprey telencephalon (delineated by dotted lines on *in toto* views). By contrast, these factors share highly similar expression patterns in other brain regions with their gnathostome counterparts. Indeed and for example, in post-telencephalic brain, expressions of lamprey COUP-TF or Dbx1 are highly similar to *Xenopus* or zebrafish COUP-TF1 ([34] and ZFIN) and zebrafish Dbx1a (ZFIN), respectively. Unlike the telencephalon, the diencephalon therefore appeared remarkably conservative in terms of patterning events (see also below, signaling systems).

In contrast, we found numerous clones expressed in the pituitary: Pitx2 (Fig. 4J–L), Six1/2 (Fig. 4D, E), Id2/3 (Fig. 3F–H), and Ldb3 (Fig. 4N) were strongly expressed in the pituitary placode and anlage at various stages, and allowed following the development of this structure, from the early stages when close apposition between basal diencephalon and oral epithelium is seen (Pitx2 at st.24, Fig. 4J), to later stages when the various elements involved are clearly individualized (st.26 for Pitx2, Six1/2, Id2/3; Figs. 4K, 4E and 3H), and finally to the development taken by the organ at early ammocoete stage (Ldb3 at st.27, Fig. 4N). Such a representation of “pituitary genes” supports with developmental data the idea that this organ and its associated neuro-secretary functions is evolutionary ancient [35], [36], [37], and that a number of specification cascades and genetic networks were already fixed in the last common ancestor of gnathostomes and lampreys for its development.

Our gene/cDNA collection also illustrated the shared genetic processes involved in dorso-ventral patterning of the neural tube in lampreys and jawed vertebrates: Pax3/7 (Fig. 4G and see also [12], [38]), Dbx1 (Fig. 4H, I), or Id2/3 (Fig. 3G) showed longitudinal expression domains which clearly correspond to D/V subdivisions of the brain such as the roof plate (Pax3/7 posterior to the di-mesencephalic boundary; Id2/3 in its post-mhb domain) or specific progenitor zones which course through the longitudinal brain axis (Dbx1 in its post-mhb domain).

As we have previously reported the analysis of LIM-homeodomain transcription factors in lamprey brain [12], [39], a special comment should be done concerning Ldb/Clim cofactors of LIM-homeodomain, for which we found two independent and distinct clones. Figure 4 (M, N) shows one of them, identified as Ldb3, expressed in a highly specific and restrictive manner in the somites up to stage 26, and then in the pituitary at amnocoete stage (described above). This is again well-conserved with pituitary development in other vertebrates, as the Ldb cofactors were originally discovered for their interactions with pituitary specifying LIM-homeodomain factors [40]. The other Ldb/Clim clone corresponded to Ldb1/CLIM1 (Figure S4, H) and displayed a ubiquitous type of expression (not shown), thereby ensuring that LIM-hd factors can be recruited to their target promoters to exert their transcriptional regulatory effects throughout the embryo.

A short special mention should be added for a small list of clones expressed in neural crest, and therefore interesting in terms of ectodermal tissue patterning. Sauka-Spengler and collaborators [41] have recently described lamprey neural crest gene regulatory network, and several clones found in our database confirmed their results, such as NeuroD2 (Fig. 3E), ZicA (not shown), or Sox genes (below).

Finally, we had the opportunity to analyze the expression of several independent and distinct members of the Sox family. Of note, those of the Sox1/2/3, i.e., B1 group have already been reported above in the “proliferation” class. There are 20 *SRY*-related high-mobility-group box (Sox) transcription factors in mammals, falling into 8 groups. Their HMG-box domain fulfills the function of DNA-binding, with the peculiarity that it binds DNA in the minor grove (reviewed in [18]). As a whole the Sox family controls cell fate and differentiation in a multitude of processes, with special emphasis in the development of the brain and neural crest [18], [42]. We found clones for two members of the SoxE class (Sox8/9/10 genes, with critical role in neural crest development). One of them, orthologous to *Petromyzon* SoxE2 recently isolated by McCauley and Bronner-Fraser [43] , was prominently expressed through neural tube, and in post-migratory and condensing neural crest cells (Fig. 5A, B). Of note, another lamprey Sox clone with unclear orthology showed the same type of expression pattern (not shown). In addition, another SoxE group member orthologous to *Lenthenteron japonicum* SoxE3 isolated by Ohtani et al. [44] and putatively identified as Sox8 (Figure S5) displayed a striking regionally-restricted, banded pattern in the forebrain, but with no detectable expression in the neural crest (Fig. 5C, D).

Among the SoxD group (Sox5/6/13 genes, with highly variable roles such as in skeletal, neural crest, cardiac, glial, or erythrocyte development), we retrieved 2 clear lamprey members, probably resulting of a lamprey-specific duplication (Figure S5, D, E). One clone displayed a complex banded forebrain (Fig. 5E) and typical neural crest pattern (arrows on Fig. 5E, F), and was highly expressed at st.24 but virtually unexpressed at st. 26 (not shown). Its paralogous SoxD member, the *Lampetra fluviatilis* orthologue of the recently isolated *Lenthenteron japonicum* SoxD1 [44], had similar expression in the forebrain but not the neural crest, and was persistently expressed at st. 26 (Fig. 5G, H). Of note, three out of these four Sox genes, in addition to the B1 group members described earlier, were expressed in the developing telencephalon, suggesting that the members of this family of transcription factors were already recruited to the anterior-most aspect of the neural tube in the common ancestor of all craniates. In agnathans also, Sox genes thus appear like a crucial gene family to control nervous system development and patterning (e.g., [42]), although some function shuffling events have probably happened in the family through vertebrate evolution.

### Midline and signaling pathways

We have previously reported that lamprey Hedgehog (Hh) shows significant differences in its expression pattern when compared to gnathostomes, particularly in the forebrain, and we have suggested that such modification of midline signaling -which govern the growth and patterning of the neuroepithelium- may be a motor for forebrain evolution. Here follows a survey of our cDNA library for other signaling systems, including the Fgf (Fibroblast Growth Factor, Wnt (Wingless-Int), and pleiotrophin signaling pathways, therefore allowing a more global picture of the signaling systems at work to control the morphogenesis of the lamprey forebrain.

#### Fgf (Fibroblast Growth Factor) signaling

**Figure 6:**
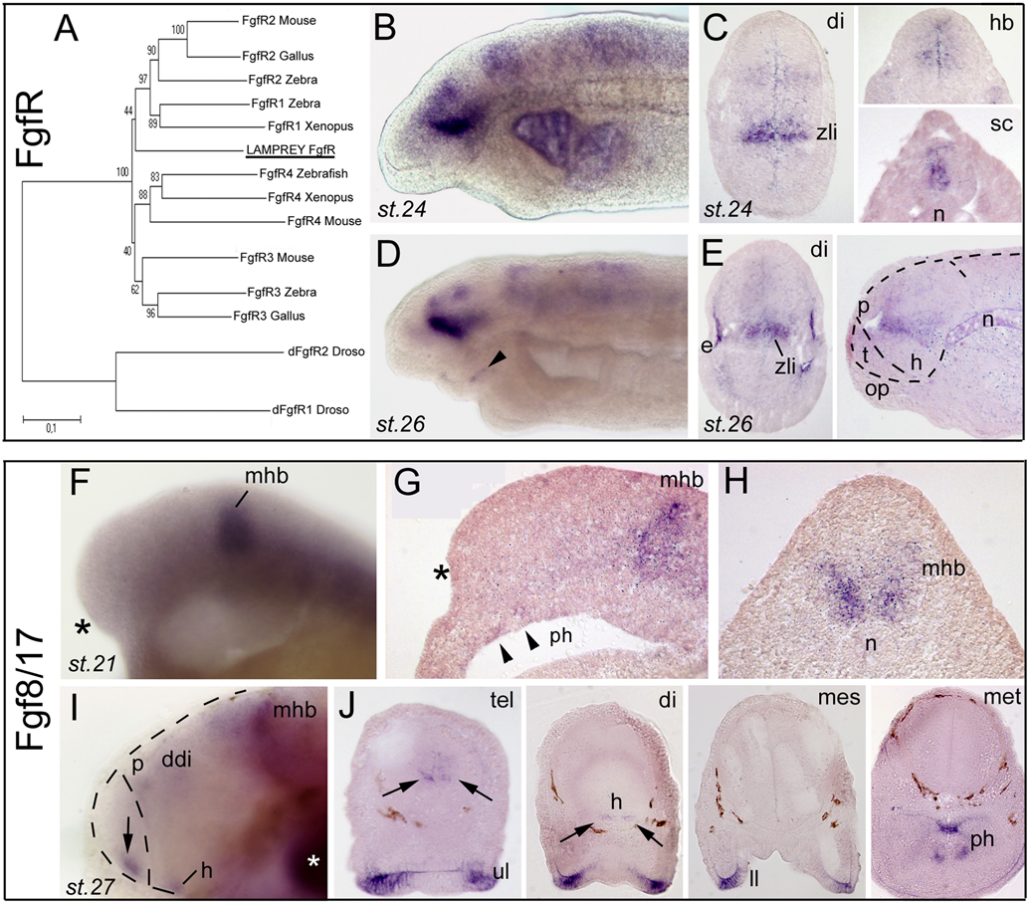
Developmental expression of Fgf signaling components in lamprey forebrain. A, phylogenetic tree (NJ) of FgfR clones. A contig was assembled out of 7 independent clones (see Table 1), and analysis clearly shows that the unique FgfR present in our database emerges at the base of the tree, and cannot be assigned a robust orthology. B,D (toto) and C,E (sections) show expression of lamprey FgfR. Toto pictures are taken from clones 15 and 83, while section pictures are taken from clones 15 and 16. The right panel in E shows a saggital section, with dotted lines delineating the brain and telencephalon. F,I (toto) and G,H,J (sections; G saggital, H and J coronal) show Fgf8/17 expression (cDNA gift from Kate Hammond [46]). The asterisks in F and G point to the absence of Fgf at rostral telencephalic level at st. 21, whereas the mhb expresses strongly the transcript. Also note faint expression in the presumptive pharynx (arrowheads). The arrows in I and J points to rostral telencephalic expression at the rostral midline at stage 27. At st. 27, strong labeling is also present in the lips and pharynx. White asterisk in I: background trapping in branchial arch. ddi, dorsal diencephalon; h, hypothalamus; p, pineal gland; ph, pharynx.

Seven independent clones for a unique FgfR were found in our library, allowing assembling a long contig for phylogenetic analysis. This FgfR case is exemplary of an often encountered situation with unresolved orthology relationships between the lamprey and gnathostome sequences (Fig. 6A, and see also figures S2 to S7). All seven clones for this FgfR showed identical expression patterns (not shown), including a prominent transverse band in the diencephalon corresponding to the zli and additional fainter expression zones in the posterior diencephalon, mesencephalon, hindbrain and spinal cord (Fig. 6B–E). At st.26, FgfR expression was also detected in the eyes (Fig. 6D) and the lips (arrowhead in Fig. 6D). Thus, lamprey FgfR pattern appears fairly similar to zebrafish FgfR3/4 but does not cover the diffuse expression throughout the forebrain (and particularly the telencephalon, noted t in Fig. 6E) as zebrafish FgfR1/2 do [45]. Concerning Fgf ligands, the only clone we retrieved from the library was not expressed at the stages examined, and we therefore analyzed the pattern of an Fgf8/17 previously isolated by Hammond and Whitfield [46] for their study of the lamprey otic vesicle. Fgf8/17 was expressed from the earliest stages examined (neurula) at the mid-hindbrain boundary in a strong and thick line (Fig. 6F). Strikingly, the anterior neural ridge/rostral telencephalic expression domain, which is also a hallmark of gnathostome Fgf8 was markedly absent, and remained absent at later stages 24 and 26 (black asterisk in Fig. 6F, G). A telencephalic Fgf8/17 domain could only be detected at stage 27, at the rostral tip of the telencephalon, together with additional expressing zones in the hypothalamus and dorsal diencephalon (Fig. 6G, H), which recapitulates the archetypal gnathostome “early” Fgf8 pattern. This finding of a putative significant heterochrony in telencephalic Fgf8/17 pattern was unexpected since a true telencephalon, subdivided in pallial and subpallial components, is clearly present (although poorly developed) at these stages. This raises the possibility that at early stages, the signaling mechanisms, which involve the Fgf8/17 secreting anterior neural ridge as one key signaling center in jawed vertebrates [47], may substantially differ in the lamprey. However, it remains equally possible that an as yet unidentified paralogous form may be present in the lamprey and expressed prior to stage 27. For instance, in zebrafish, Fgf3 early expression and function are partially redundant with those of Fgf8 [48] and an Fgf8 paralog, Fgf19, was demonstrated to play a role in ventral telencephalic and diencephalic patterning [49]. An exhaustive characterization of the Fgf family in lamprey based on genomic data will be crucial to assess the possibility of similar expression and function shuffling processes in the Fgf family in the lamprey.

#### Wnt (Wingless-Int) signaling

Our database was rich in Wnt pathway components, allowing a thorough comparative analysis of ligands, receptors, and antagonists of this crucial pathway for forebrain specification and development (Figures S6 and S7 for phylogenetic analyses).

**Figure 7:**
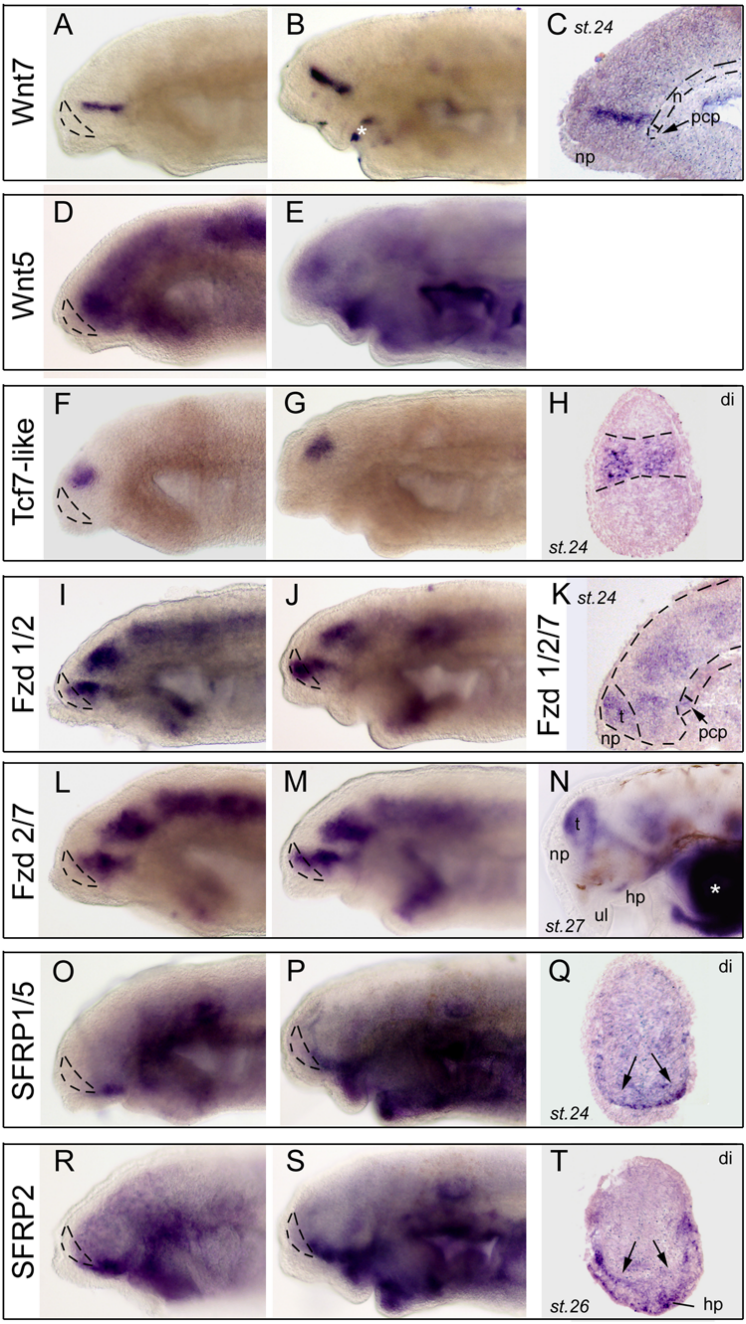
Developmental expression of Wnt signaling components in lamprey forebrain. A,B (toto) and C (saggital section) show Wnt7 expression pattern (clone 70). In C the arrow points to the prechordal plate (pcp) which does not express Wnt7. D,E, expression of Wnt5 in toto (clone73). F,G (toto) and H (section) show Tcf7-like (clone 24) expression as a very discrete and sharp domain in the diencephalon. I,J (toto) show expression of Frizzled 1/2 (I shows clone 101 and J shows clone 94). K (saggital section) shows another Frizzled 1/2/7 member (clone 100). Dotted lines delineate the brain, telencephalon and notochord, and the arrow points to the Fzd-expressing prechordal plate (pcp). L,M,N show in toto views of Frizzled2/7 (clone 95) through stage 27. O,P (toto) and Q (section) show SFRP1/5 expression. O and Q show clone 103 whereas P shows clone 102. R,S (toto) and T (section) show SFRP2 expression. R and T show clone 97 whereas S shows clone 96. On whole-mount pictures, dotted lines delineate the telencephalon. Arrows in Q and T indicate basal hypothalamic expression. White asterisks indicate background trapping in the mouth or branchial arches. np, nasal placode; hp, hypophyseal placode.

Concerning Wnt ligands, the library contained 4 independent clones for a Wnt7 and a Wnt5 lamprey member, respectively (Fig. 7A–E). Both Wnt factors showed a nested expression as a sharp transverse band through the diencephalon corresponding to the zli (compare to FgfR above or Hh in [12]), at st.24 as well as at st.26. The Wnt5 clone additionally presented a low level of expression throughout the neural tube (Fig. 7D, E). Thus, it appears that Wnt ligands are preferentially and highly secreted from a mid-diencephalic signaling centre in lampreys, which is consistent with conservation in lamprey of the essential role of Wnt signaling in promoting diencephalic identity (e.g., [50]). This conservation is strongly supported by the expression of Tcf7 as a sharp and large domain restricted to the diencephalon (Fig. 7F–H), that is in an exactly identical manner to the Lef-Tcf factors downstream of Wnt signaling in other vertebrates (e., g., see ZFIN for zebrafish).

We next retrieved a large number of clones (10 total) for the Wnt receptors Frizzled. Three of them showed a typical banded pattern, with two main transverse expressing zones located just rostrally and just caudally to the Wnt-producing zli. They are shown in Fig. 7I–N and they all belong to the Frizzled 1/2/7 orthology class in the Frizzled receptor superfamily (Figure S7). Lamprey Frizzled 1/2/7 expression additionally encompassed the telencephalon (Fig. 7I, J, K, N), and was also present in the prechordal plate, a vertebrate-specific, rostral-most “extension” of the notochord with important signaling properties and particularly crucial for the development of the hypothalamus (Fig. 7K, arrow). Although the frizzled members in mouse [51] or zebrafish (ZFIN database) show less spectacular and more widespread patterns than those found here in lamprey embryos, they were reported absent from the Wnt-secreting, zli region (see summary figure). Likewise, the zebrafish prechordal plate also expresses Frizzled receptors [52]. In summary, these similarities suggest that the main source of Wnt ligand in the zli signals through Frizzled receptors both anteriorly and posteriorly in the diencephalon, in the lamprey as in other vertebrates.

SFRPs (Secreted Frizzled-Related Protein) are crucial components of the Wnt pathway, as they act as modulators or antagonists of Wnt signaling (reviewed in [53]). Here we present the expression patterns of lamprey SFRP1/5 (2 independent clones) and SFRP2 (4 independent clones) which, importantly, clearly belong to the group 1, 2, and 5 of SFRPs after phylogenetic analysis (Figure S7). Both lamprey SFRPs showed remarkable and almost identical patterns, being expressed exclusively as a sharp and restricted domain in the basal diencephalon, at the base of the hypothalamus (Fig. 7O–T). None of these SFRPs were transcribed in the telencephalon (Fig. 7O, P and 7R, S). This contrasts with the results obtained in gnathostomes in which an inhibition of Wnt signals by SFRPs (particularly SFRP1/SFRP5 and Tlc in zebrafish) is known to be required to specify and promote the telencephalic territory [54], [55], [56]. A schematic comparison with zebrafish, where the Wnt pathway has been thoroughly studied is given on the summary Figure (schematized from data from ZFIN and [56]; the situation in tetrapods is highly similar to zebrafish, e.g., [57]). It suggests that the major difference in Wnt pathway distribution between the two species may be related to the absence of Wnt inhibitors/antagonists expression in the developing telencephalon of lampreys, at least up to stage 26. A more thorough analysis at later stages will be necessary to further address this point, but it is attractive here to relate the lack of SFRPs to the tiny size (and possibly the fewer subdivisions, although there are no functional data available from model organisms to support this to our knowledge) of the telencephalon in embryonic lampreys. In this hypothesis, the lamprey could exemplify the evolutionary consequences of changes in the spatio-temporal expression of the modulators of a signaling pathway –i.e., not the ligand itself- on the anatomy and morphogenesis of a structure.

#### Midkine signaling

**Figure 8:**
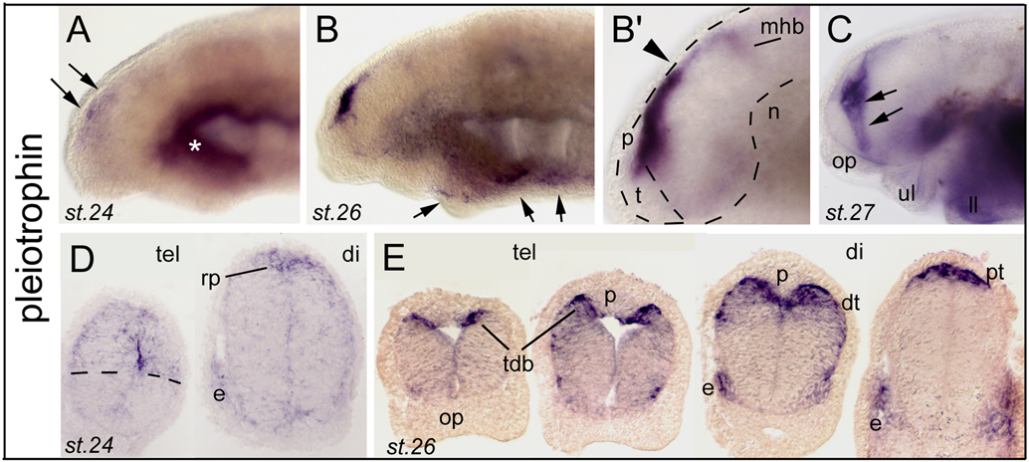
Developmental expression of pleitrophin (Ptn) in lamprey forebrain. A to C (toto) and DE (sections) show Ptn expression. AB show clone 161 and B'CDE show clone 163. Note that expression is diffuse at stage 24 (A, D) and condenses at the dorsal midline at stage 26 (B, B') and 27 (E). Arrows in A point to dorsal telencephalic and dorsal diencephalic expression. Arrows in B indicate a thin line of expression in lower lip (ll) and ventral aspect of the branchial arches. In B' and D, rp indicates the roof plate. Note the difference of expression level at the di-mesencephalic border (arrowhead in B') between the pretectum (pt) and the tectum. In C, the arrows point to the Ptn-expressing tel-diencephalic boundary. E shows an antero-posterior series through the telencephalon and tel-diencephalic boundary, highlighting the relationship of the Ptn pattern and the suspected morphogenetic movements in the vicinity of the pineal gland (p). tdb indicates the telencephalo-diencephalic boundary region. rp, roof plate; dt, dorsal thalamus; p, pineal gland; pt, pretectum.

Pleiotrophin (Ptn) is a secreted heparin-binding growth factor and developmentally-regulated cytokine with properties related with tumorigenesis, and is also involved in neural development where it controls neurite outgrowth or neuronal migration. Together with MK (midkine), Ptn belongs to the Midkine family -which is thus formed by only two members. Zebrafish and *Xenopus* MK can induce neural tissues, and both MK and PTN are localized in the radial glial processes of the mouse embryonic brain (reviewed in [58]). In the present study, we found four independent clones for a lamprey pleiotrophin, which unexpectedly showed a spectacular dorsal midline type of expression profile (Fig. 8; see also Figure S8 for phylogeny). This expression was somehow diffuse at st.24 in the dorsal-most aspect of the anterior neural tube and particularly the dorsal telencephalon (Fig 8A, D), and became strong and heavily concentrated towards the telencephalic/diencephalic dorsal midline at st. 26 (Fig. 8B, B'), also highlighting the previously mentioned typical fork pattern of the dorsal thalamus and di-telencephalic boundary (Fig. 8E, compare with PCNA for example). At st.27, expression spread to the telencephalic/diencephalic boundary zone (Fig. 8C, arrows). Notably, the roof plate of other studied vertebrates does not express Ptn or Mk. In zebrafish, Mk1 and Mk2 are rather expressed in the dorsal-most or ventral-most parts of the ventricular neuroepithelium, but are excluded from the roof or floor plate, respectively [59], while Ptn is strongly expressed in the floor plate (Zfin). On the other hand, mouse Ptn is expressed in the dorsal half of the neural tube but not the roof plate (Fan et al., 2000). As stressed out by Winckler and colleagues [59] it appears that the expression and function of these midkines are highly divergent among vertebrates (mouse, *Xenopus*, chick and fish). Our present findings in agnathans reinforce this trend, and further suggest that this signaling pathway is highly versatile and may be recruited to very divergent processes, implicating also that the constraints on its regulation of expression must be surprisingly weak. In this frame, we would like to stress that from a morphogenetic point of view, these differences in Ptn expression among vertebrates are interesting to relate to the highly divergent cell movements involved in telencephalic formation: evagination, more or less pronounced, in tetrapods; eversion in teleost fishes; or partial evagination in lampreys. The dorsal midline is thought to be instructive in directing these movements. The expression of lamprey Ptn being unique in the tel-diencephalic roof plate may underlie the singular growth and morphogenesis of its dorsal telencephalon.

### Conclusions

**Figure 9:**
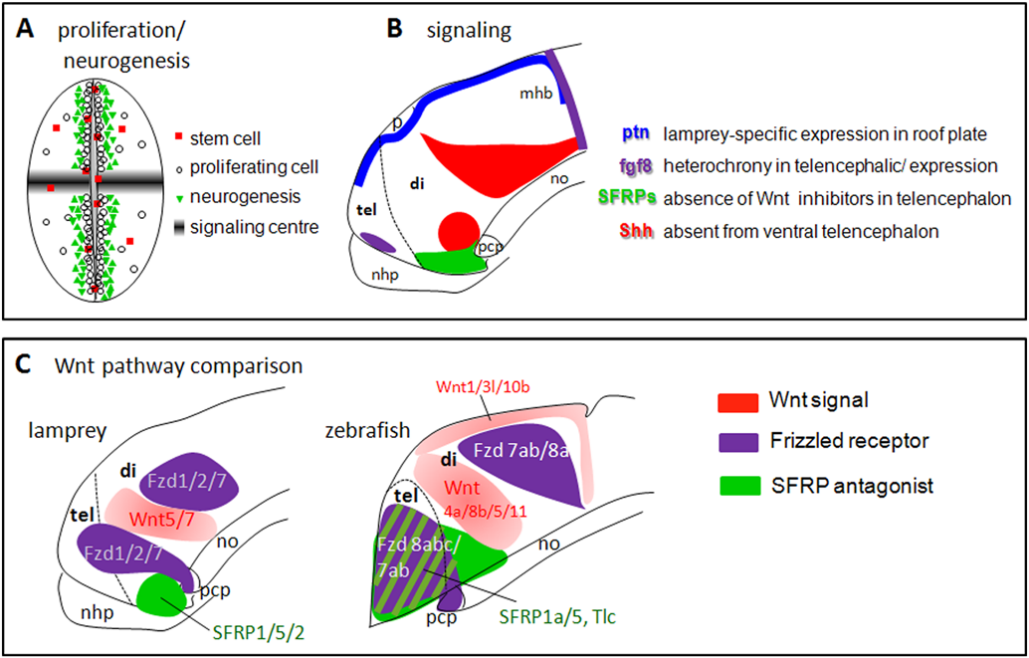
Conserved proliferation and neurogenesis processes, but divergent signaling mechanisms in the forebrain in lampreys and gnathostomes. A and B summarize the main findings of this study. In A, the neurogenesis pattern schematized is highly comparable to what has been found in jawed vertebrates. By contrast, in B, four major signaling systems which control the growth, the patterning, and the morphogenesis of the forebrain are depicted, and the type of difference when compared to jawed vertebrates is indicated. C provides a more detailed comparison of the Wnt signaling pathways in embryonic lampreys (present data) and in zebrafish (compiled from ZFIN and [56]). It should be noted that the absences of expression we observed for signaling systems in telencephalon await for evidences from functional and/or complete genomic data that these apparent lacks are not compensated by other family members.

The expression analysis of 43 genes involved at various steps of (fore)brain development in lampreys provides a global picture of forebrain embryogenesis in this agnathan species, and a better understanding of the mechanisms which have allowed the emergence of the “craniate” type of forebrain (Fig. 9A–C).

As stated in introduction, a major evolutionary novelty in the brain of craniates is the presence of a forebrain, including a true telencephalon and a diencephalon. It is striking to see that in the lamprey lineage the developmental genetic networks which are responsible for specification and patterning of the diencephalon (including the pituitary) are well established, leading to the formation of a typical “craniate-type” diencephalon, organized into transverse subdivisions, and with the zli organizer having a suspected crucial role in the establishment of this organization. At the difference of the diencephalon which appears very “conservative”, we believe that major insights on the diverse mechanisms which may have been used in the course of evolution for telencephalic evolution emerge from our comprehensive survey. We propose two main and probably strongly inter-related mechanisms: (1) the *de novo* expression and recruitment of regional specification genes in the anterior-most part of the alar plate (from which the telencephalon is exclusively derived). In embryonic lamprey telencephalon, orthologues of gnathostome telencephalic-expressed genes such as COUP-Tf, Dbx1, Six1/2 or Id2/3 were not transcribed. This confirms and extends previous reports using candidate approach strategy, where a typical ventral telencephalic specification factor such as Nkx2.1 was found unexpressed in this forebrain region in lampreys [11], [12]. We suggest that the recruitment of some new factors to pattern the gnathostome telencephalon originates at least in part in upstream signaling events, i.e., (2) a strong contribution of signaling systems, whose components appear to be significantly modified both in space and time between lampreys and gnathostomes. Extending our previous findings on the Hedgehog pathway, we provide here new examples of potential heterochronies (Fgf8/17 pathway) or differential modulation of signaling (SFRPs in the Wnt pathway) or else totally different patterns (pleiotrophin signaling) which may have deep impacts on lamprey telencephalic patterning and morphogenesis (Fig. 9B). Our findings highlight the notion that morphological changes rather occur by modifying gene regulation than sequence (see [60]) and open interesting and new perspectives in the field of the study of lamprey regulatory sequences and comparative genomics.

As opposed to these signaling and patterning divergences observed in the telencephalon, our survey clearly indicates that the basic molecular mechanisms for proliferation and neurogenesis as well stemcellness throughout the neural tube are shared among craniates (Fig. 9A). This finding was relatively expected, as the discussed factors are recruited to cellular processes which appear to be common to all deuterostomes, and even more so, have been suggested to reflect conserved cell type differentiation in the last common bilaterian ancestor [61]. It is nevertheless interesting to observe, in lampreys, the presence of neurogenic zones characterized in the anteroposterior axis of the forebrain by patches of proliferation/neurogenesis, and which are separated by low/no proliferation zones, often corresponding to signaling centers locations (Fig. 9A). This mode of forebrain development is typically “vertebrate-like” and was therefore recruited in the last common ancestors of craniates.

## Methods

### cDNA library, sequencing and phylogenetic analyses

Three lamprey cDNA libraries (embryonic-prolarval (st. 20–26) and adult brain and eye in *Lampetra fluviatilis* or larval to post-metamorphic heads in *Petromyzon marinus*) were constructed in pSPORT1 vector using the directional cloning protocol of the Superscript plasmid system with Gateway technology (InVitrogen). They were plated, arrayed robotically and submitted to large-scale EST sequencing on an ABI3730xl by the Genoscope (Evry, France). Sequencing was conducted using the reverse universal primer, which generates sequences in the 5′ region of inserted cDNA fragment. The global gene content of the cDNA database thus generated will be reported elsewhere. Searches for genes involved in forebrain formation were achieved by a candidate gene approach, using BLASTN searches of our lamprey cDNA database with zebrafish sequences as queries. The identity of the hits was first confirmed by a reverse BLASTN analysis on Genbank. Orthology relationships were further assessed by phylogenetic analysis (shown in supplementary figures or available on request). For this purpose, lamprey sequences were aligned with family members in other species, and phylogenetic trees were constructed with the Neighbor Joining method using MEGA4.0.

In cases when both *L. fluviatilis* and *P. marinus* sequences were retrieved for a single gene, a nucleotide identity comprised between 98% (e.g., between clones 19 and 64 for Dbx1) and 100% (e.g., between clones 113 and 77 for Pitx2, see Table 1) was observed on overlapping fragments, suggesting a close relationship between the two lamprey species. This conclusion was also supported by the expression analysis, since identical, highly specific signals were reproducibly obtained on *P. marinus* embryos with homologous and heterologous probes (see figure legends and Table 1 for clone details).

Sequences for the *L. fluviatilis* and *P. marinus* sequences analyzed in this manuscript have been submitted to Genbank and correspond to accession numbers FP243278 to FP243359.

### *In situ* hybridization

Selected clones were picked from the arrayed cDNA library, and checked for insert presence and size by restriction digestion (BamH1+EcoR1digest). The insert was then PCR-amplified using T7 and Sp6 primers, and 1–10 ng of the PCR product was used as template for digoxygenin-labeled RNA probe synthesis using digoxygenin 11-UTP (Roche) and Sp6 RNA polymerase (Promega) following standard protocols. Labeled RNA probes were purified on NucleoSpin RNAII columns and stored in 50%formamide at −80°C until use. *In situ* hybridization was carried out using an automat (Intavis *InsituPro* VS) in the following conditions. Briefly, *P. marinus* embryos/prolarvae/larvae were rehydrated, bleached (6%H_2_O_2_, 1 h), permeabilised by proteinase K treatment (10 µg/ml, 45 min), and postfixed (4% paraformaldehyde, 20 min). Pre-hybridization and hybridization medium contained 50% formamide, 5XSSC, 2% blocking powder (Boehringer), 50 µg/ml heparine, 0.1 mg/ml tRNA, 0.5 M EDTA, and 10% CHAPS. Hybridization was carried out at 70°C for 16 hours. After post-hybridization washes, embryos were incubated in blocking buffer (PBS/Triton 0.1% containing 15% serum and 2 mg/ml BSA) for 3 hours at 4°C before addition of the alkaline phosphatase coupled anti-digoxygenin antibody (1∶1500, Roche) for 12 hours at 4°C. After washes, color reaction was performed in the presence of NBT/BCIP (Roche). For *in toto* observation of expression patterns, embryos were dehydrated and cleared in benzyl-benzoate before mounting in Entellan. Otherwise they were dehydrated through ethanol and butanol steps, paraffin-embedded and sectioned (8 µm thick) on a microtome.

Photographs were taken on a Nikon E800 microscope equipped with a Nikon Dxm1200 camera, and mounted for figures with Adobe Photoshop. Images were corrected for brightness and contrast only.

All the patterns shown were obtained in *P. marinus*, using either homologous or heterologous probes from the closely related *L. fluviatilis* species. In the latter case, highly specific patterns were always observed and we directly checked for pattern identity between the two species by a parallel analysis in *L. fluviatilis* for several probes (Otx and LIM-homeodomain). Stages were determined according to the staging table established for *L. reissneri* by Tahara [62].

## Supporting Information

Figure S1 High power magnification photographs through the diencephalon of embryonic lampreys, showing the distribution of transcripts for the “proliferation class” of clones reported in Figs 1 and 2, in the depth of the neuroepithelium (VZ, SVZ, and MZ). Gene names are indicated.
(4.63 MB TIF)

Figure S2 Phylogenetic analysis of “proliferation/stem cell” class of clones. A, clone 140 (identical to clones 31, 131, 132, smaller clones included into cl.140), shown in Figure 1, corresponds to lamprey PCNA. B, clone 40, shown in Figure 2AB, corresponds to a lamprey Msi2 ortholog. C, clone 49, shown in Figure 2CDE, corresponds to a lamprey Notch with non-defined orthology relationship towards the 3 Notch classes found in gnathostomes. D and E, clone 48 and 133 (clone 48 is shown in Figure 2FGH) are two lamprey Delta. Clone 48 belongs to the Delta 1 class of orthology with good support, whereas clone 133 shows poorly resolved orthology relationship. F, clone 168 = 169 (clone 169 is shown in Figure 2I–K) is a lamprey pisolo. Pisolo is a DUF1279 domain containing factor (identified in medaka fish by Alunni and Joly, personal communication). Accession numbers for pisolo genes are as follows: Homo, AK055618; Gallus, AJ719368; Mouse, AK162396; zebra chr16, BC090760; zebra chr19, XM_001918550; Aedes, XM_001655586. The outgroup is composed of a zebrafish, a chicken, and a human DUF1279 domain containing ORF, the three of them corresponding to ORFs of unknown function and representing the closest-related genes to pisolo.
(0.57 MB TIF)

Figure S3 Phylogenetic analysis of “neurogenesis” class of clones. A, clone 88, shown in Figure 3AB, corresponds to a lamprey Neurogenin1. B, clones 18 (shown in Figure 3CDE) correspond to a lamprey NeuroD2. C, clones 29 = 123 = 125 = 127 (clones 123 and 127 are shown in Figure 3FGH) correspond to a lamprey Id2/3 with good support, but the orthology between Id2 and Id3 remains unresolved.
(0.47 MB TIF)

Figure S4 Phylogenetic analysis of “patterning” class of clones. A, clone 22, and B, clone 25 (clone 25 is shown in Figure 4C) both correspond to a lamprey COUP-Tf/NR2F (Nuclear Receptor subfamily 2). The two clones are from Lampetra (see Table 1) but their sequence does not overlap. However, the fact that they are both NR2F members with poorly supported orthology towards the subfamily 1 or 2 probably suggests that they correspond to the same gene. The fact that they show identical expression patterns (clone 22 not shown) also supports this idea. C, clone 114, shown in Figure 4F, corresponds to a lamprey LIM-kinase 2. D, clone 20, shown in Figure 4DE, clearly corresponds to a lamprey Sox1/2 factor. The available sequence fragment does allow resolving properly orthology within the Six1/Six2 group. E, clone 77 (and 7 other clones, see Table 1) from Lampetra and clone 113 (from Petromyzon) correspond to lamprey Pitx2. Clone 113 is shown in Figure 4JKL and is identical in sequence to Petromyzon PitxA published in Genbank. F, clone 19 = 64 (clone 19 is shown in Figure 4HI) correspond to lamprey Dbx1. G, clone 17, shown in Figure 4B, corresponds to a lamprey FoxB1. H, clone 8 (ubiquitously expressed, not shown, see text) is lamprey Ldb1 (Ldb, LIM Domain Binding protein, previously called CLIM for Cofactor of LIM). I, clone 160, shown in Figure 4MN, is a lamprey Ldb3. Ldb3 subgroup of Ldb is strongly divergent from Ldb1/Ldb2, in that it contains a PDZ domain, a Zasp motif, and 3LIM domains. Clustal alignment is shown with other Ldb3 members, and shows very high conservation of the available lamprey sequence (shown entirely) through the whole N-terminal part of the protein, encompassing the PDZ domain and the Zasp motif (short 26 a.a. motif).
(1.16 MB TIF)

Figure S5 Phylogenetic analysis of Sox family member clones. A, clones 28 = 137, shown in Figure 2LL', correspond to a lamprey Sox3. B, clones 38 = 42, shown in Figure 2LMN, correspond to a clear SoxB1 (Sox1/2/3) lamprey factor, but with poorly supported orthology relationship. C, C', clones 115 = 117 = 120 and clone 37, shown in Figure 5A–D, correspond to two lamprey SoxE (Sox8/9/10) members. The former is Sox8 and the latter has again a non-resolved orthology relationship. C' shows a tree including only lamprey SoxE group members, and allows to propose orthologies with previously isolated Japanese lamprey and Petromyzon SoxE2 and SoxE3. D and E, clones 116 and 122, shown in Figure 5E–H, are two lamprey group D (Sox5/6/13) members. They share a 79 amino-acid long identical stretch of sequence, which corresponds to their dimerisation domain, a functional domain which is unique to the SoxD group. Clustal alignment with other vertebrate SoxD members at this level is show in D. The NJ tree in E shows that the two lamprey paralogs emerge at the root of the tree, and may correspond to a lamprey-specific duplication.
(0.94 MB TIF)

Figure S6 Figure S6: phylogenetic analysis of Wnt pathway clones. A, clones 70 = 1 = 2 (clone 70 is shown in Figure 7ABC) are lamprey Wnt7. B, clone 73, shown in Figure 7DE, corresponds to lamprey Wnt5. C, clone 24, shown in Figure 7FGH is a lamprey Tcf7-like whose orthology is not robustly supported between the Tcf7-like1 and Tcf7-like2 groups.
(0.84 MB TIF)

Figure S7 Phylogenetic analysis of Wnt pathway clones, continued. A, clones 94 = 101 = 105 = 107 = 108 (clone 101 is shown in Figure 7I and clone 94 is shown in Figure 7J) are lamprey Frizzled 1/2. Note the organization of the Fzd superfamily, with 4 large subgroups, namely groups Fzd1/2/7, Fzd 5/8, Fzd 4/9/10, and Fzd 3/6. B, clones 100 = 136 (clone 100 is shown in Figure 7K) are also in group Fzd1/2/7, with no support towards one of the 3 possible gnathostome orthologies. Clone 95 (tree not shown, in situ hybridization presented in Figure 7LMN) is in the same case, and identified as an Fzd2/7 member. As a result of this analysis of Fzd clones, we conclude that there are (at least) 3 distinct lamprey group Fzd1/2/7 members, like in gnathostomes, although their exact orthology relationships are uncertain (see also Table 1). C, clones 96 = 97 = 98 = 109 (96 and 97 are shown in Figure 7RST) correspond to lamprey SFRP2. D, clones102 = 103 (both shown on Figure 7OPQ) correspond to a lamprey SFRP1/5, without robust support toward SFRP1 or SFRP5 orthology.
(0.69 MB TIF)

Figure S8 Phylogenetic analysis of pleiotrophin clones. The tree shows that clone 161 (from Petromyzon) and clones 162 = 163 = 164 (from Lampetra) are lamprey midkines and probably belong to the pleiotrophin group. Clones 161 and 163 are shown in Figure 8.
(4.13 MB TIF)
