# Supplementary figures and images for: Neurodevelopment Genes in Lampreys Reveal Trends for Forebrain Evolution in Craniates
Source: PLoS One. 2009 Apr 28;4(4):e5374. doi: 10.1371/journal.pone.0005374 (PMC2671401; doi:10.1371/journal.pone.0005374)

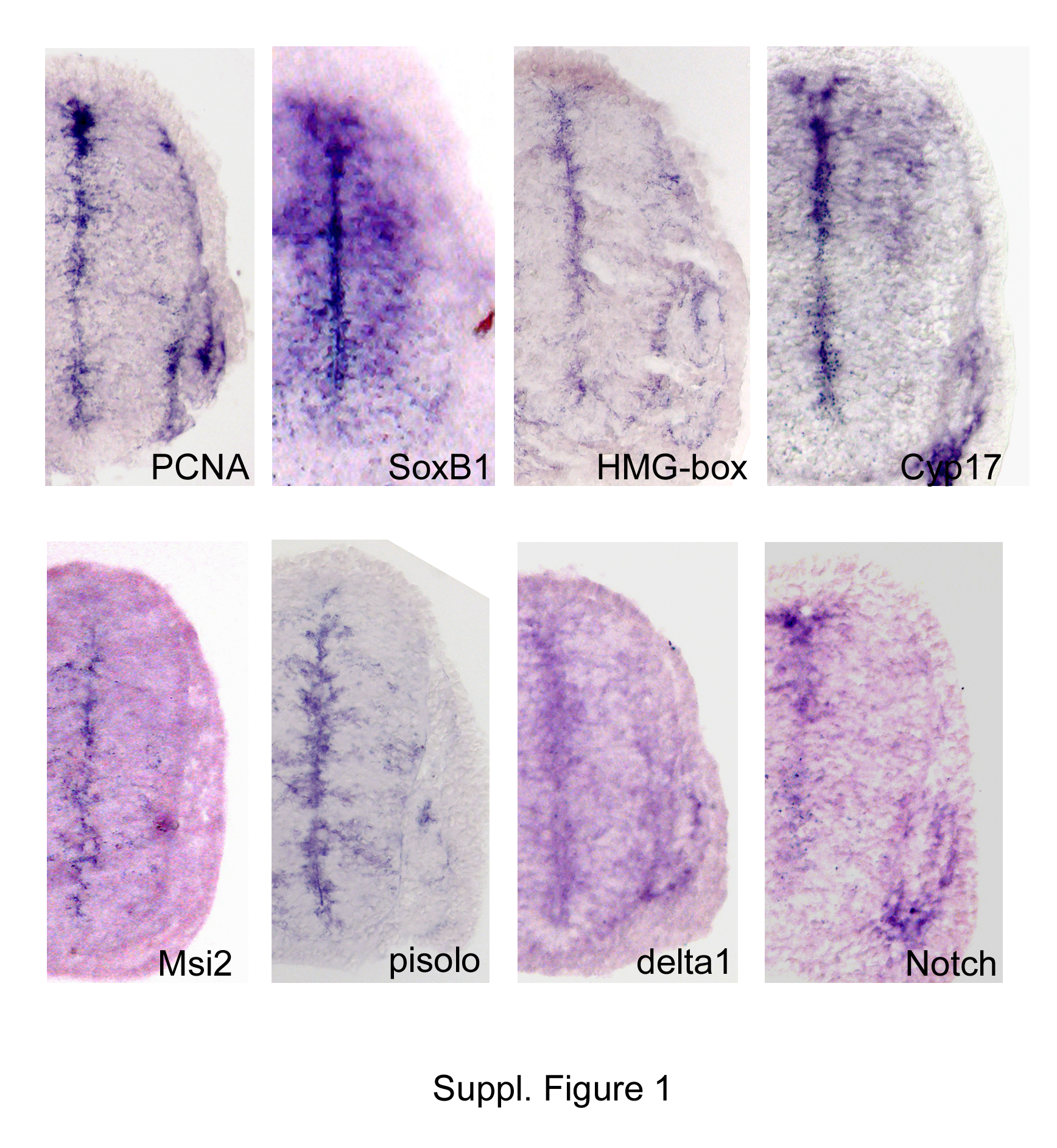

Supplement: Figure S1 — High power magnification photographs through the diencephalon of embryonic lampreys, showing the distribution of transcripts for the “proliferation class” of clones reported in Figs 1 and 2, in the depth of the neuroepithelium (VZ, SVZ, and MZ). Gene names are indicated. (4.63 MB TIF) [file pone.0005374.s001.tif]

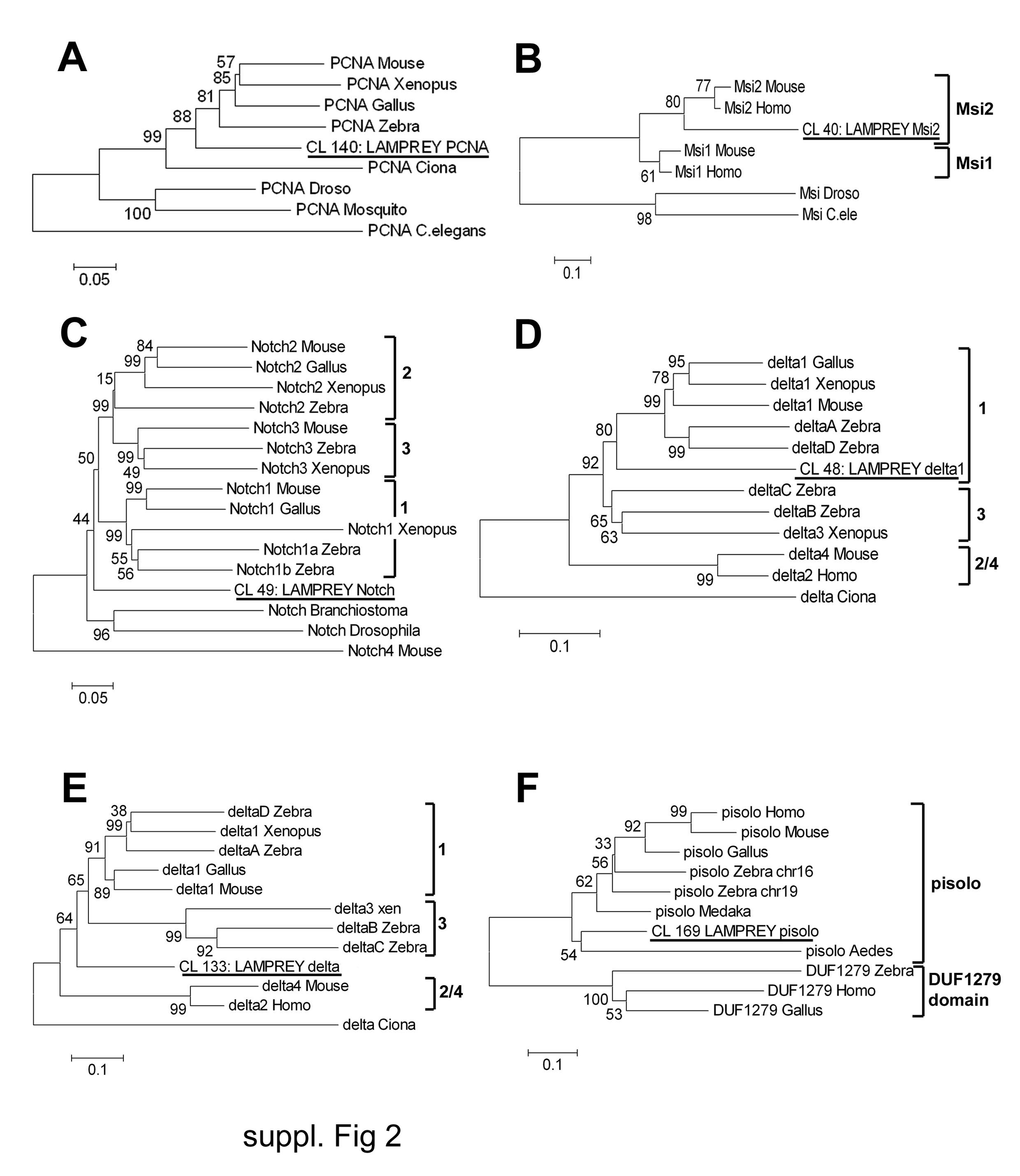

Supplement: Figure S2 — Phylogenetic analysis of “proliferation/stem cell” class of clones. A, clone 140 (identical to clones 31, 131, 132, smaller clones included into cl.140), shown in Figure 1, corresponds to lamprey PCNA. B, clone 40, shown in Figure 2AB, corresponds to a lamprey Msi2 ortholog. C, clone 49, shown in Figure 2CDE, corresponds to a lamprey Notch with non-defined orthology relationship towards the 3 Notch classes found in gnathostomes. D and E, clone 48 and 133 (clone 48 is shown in Figure 2FGH) are two lamprey Delta. Clone 48 belongs to the Delta 1 class of orthology with good support, whereas clone 133 shows poorly resolved orthology relationship. F, clone 168 = 169 (clone 169 is shown in Figure 2I–K) is a lamprey pisolo. Pisolo is a DUF1279 domain containing factor (identified in medaka fish by Alunni and Joly, personal communication). Accession numbers for pisolo genes are as follows: Homo, AK055618; Gallus, AJ719368; Mouse, AK162396; zebra chr16, BC090760; zebra chr19, XM_001918550; Aedes, XM_001655586. The outgroup is composed of a zebrafish, a chicken, and a human DUF1279 domain containing ORF, the three of them corresponding to ORFs of unknown function and representing the closest-related genes to pisolo. (0.57 MB TIF) [file pone.0005374.s002.tif]

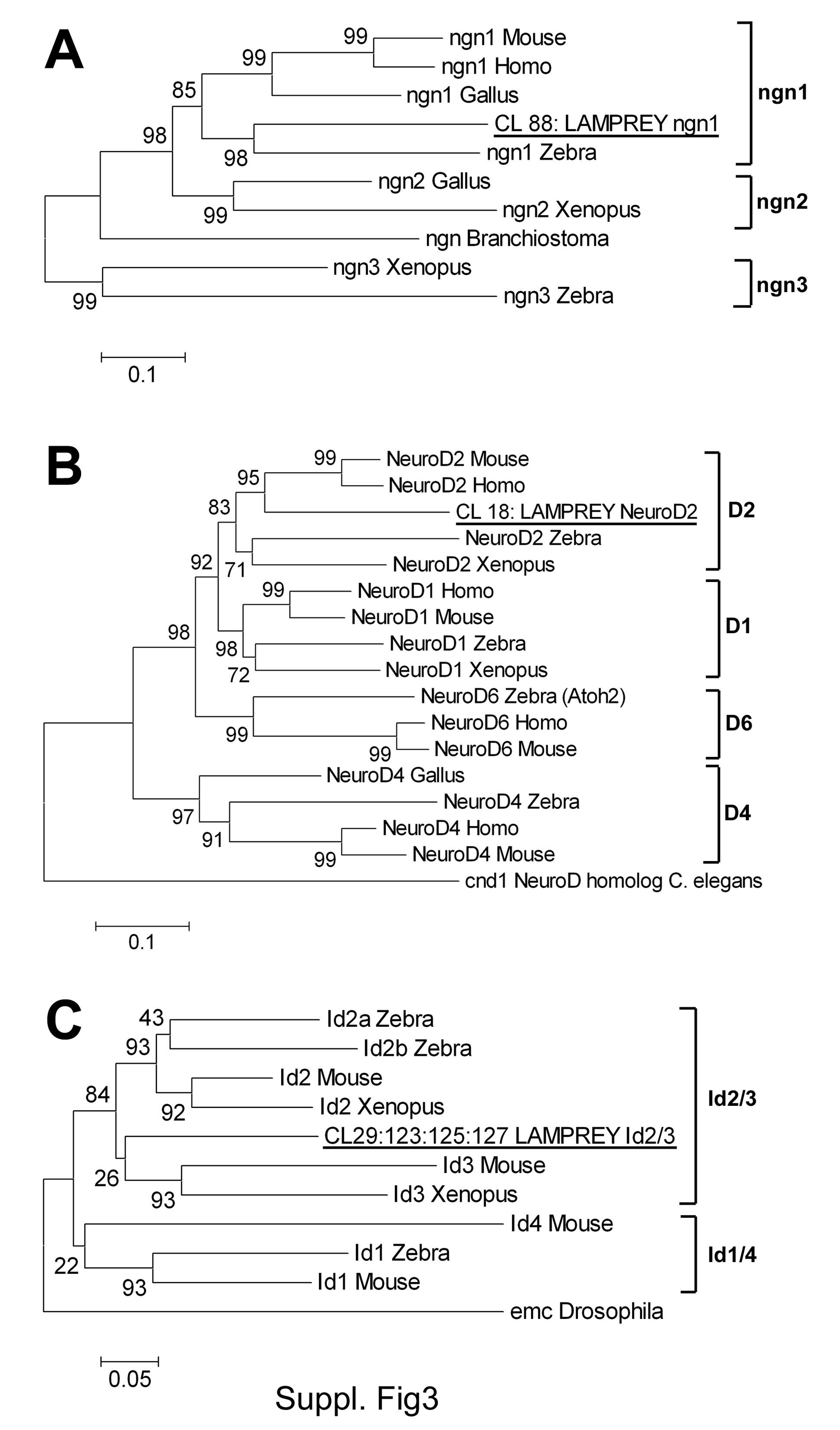

Supplement: Figure S3 — Phylogenetic analysis of “neurogenesis” class of clones. A, clone 88, shown in Figure 3AB, corresponds to a lamprey Neurogenin1. B, clones 18 (shown in Figure 3CDE) correspond to a lamprey NeuroD2. C, clones 29 = 123 = 125 = 127 (clones 123 and 127 are shown in Figure 3FGH) correspond to a lamprey Id2/3 with good support, but the orthology between Id2 and Id3 remains unresolved. (0.47 MB TIF) [file pone.0005374.s003.tif]

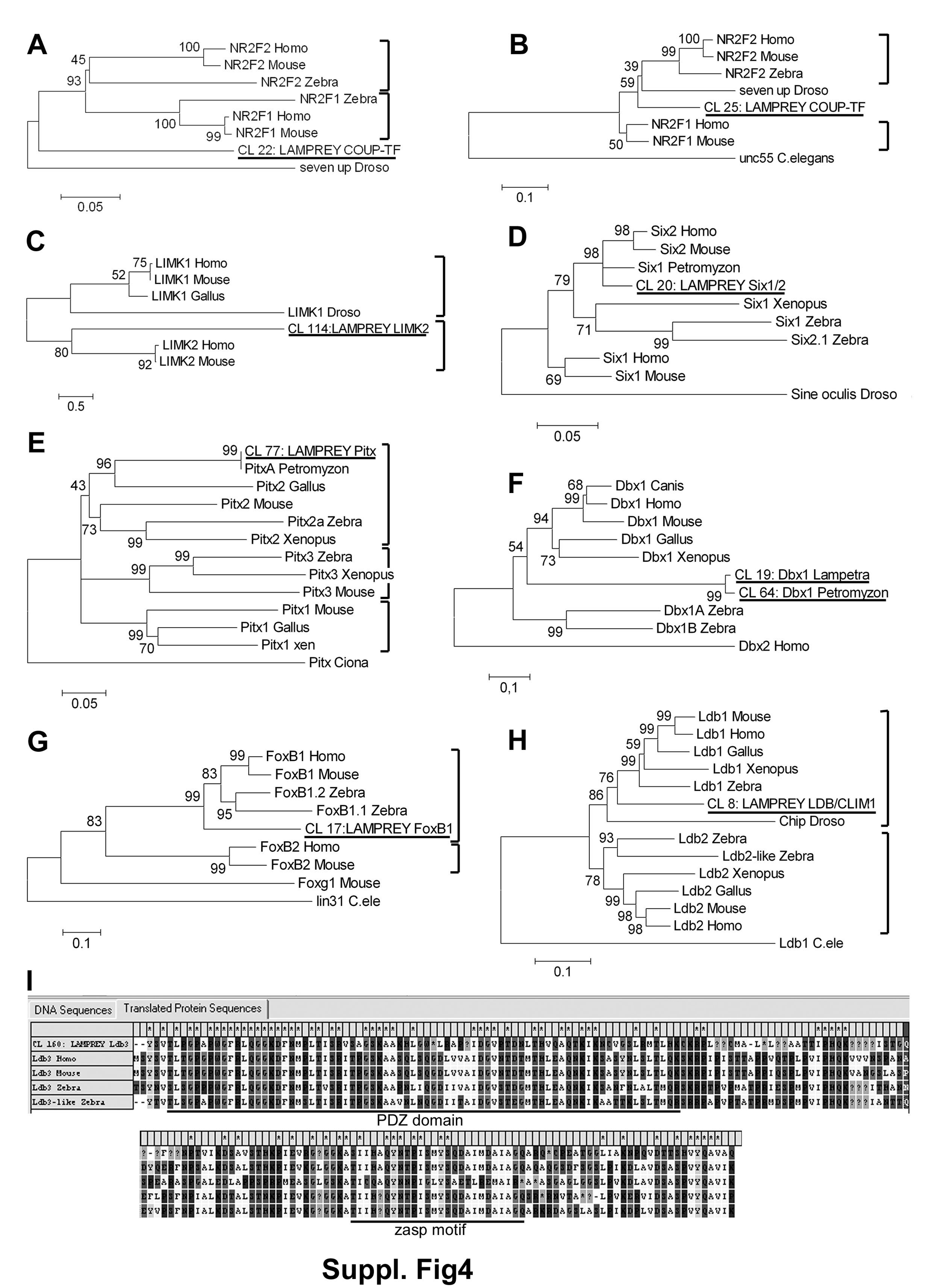

Supplement: Figure S4 — Phylogenetic analysis of “patterning” class of clones. A, clone 22, and B, clone 25 (clone 25 is shown in Figure 4C) both correspond to a lamprey COUP-Tf/NR2F (Nuclear Receptor subfamily 2). The two clones are from Lampetra (see Table 1) but their sequence does not overlap. However, the fact that they are both NR2F members with poorly supported orthology towards the subfamily 1 or 2 probably suggests that they correspond to the same gene. The fact that they show identical expression patterns (clone 22 not shown) also supports this idea. C, clone 114, shown in Figure 4F, corresponds to a lamprey LIM-kinase 2. D, clone 20, shown in Figure 4DE, clearly corresponds to a lamprey Sox1/2 factor. The available sequence fragment does allow resolving properly orthology within the Six1/Six2 group. E, clone 77 (and 7 other clones, see Table 1) from Lampetra and clone 113 (from Petromyzon) correspond to lamprey Pitx2. Clone 113 is shown in Figure 4JKL and is identical in sequence to Petromyzon PitxA published in Genbank. F, clone 19 = 64 (clone 19 is shown in Figure 4HI) correspond to lamprey Dbx1. G, clone 17, shown in Figure 4B, corresponds to a lamprey FoxB1. H, clone 8 (ubiquitously expressed, not shown, see text) is lamprey Ldb1 (Ldb, LIM Domain Binding protein, previously called CLIM for Cofactor of LIM). I, clone 160, shown in Figure 4MN, is a lamprey Ldb3. Ldb3 subgroup of Ldb is strongly divergent from Ldb1/Ldb2, in that it contains a PDZ domain, a Zasp motif, and 3LIM domains. Clustal alignment is shown with other Ldb3 members, and shows very high conservation of the available lamprey sequence (shown entirely) through the whole N-terminal part of the protein, encompassing the PDZ domain and the Zasp motif (short 26 a.a. motif). (1.16 MB TIF) [file pone.0005374.s004.tif]

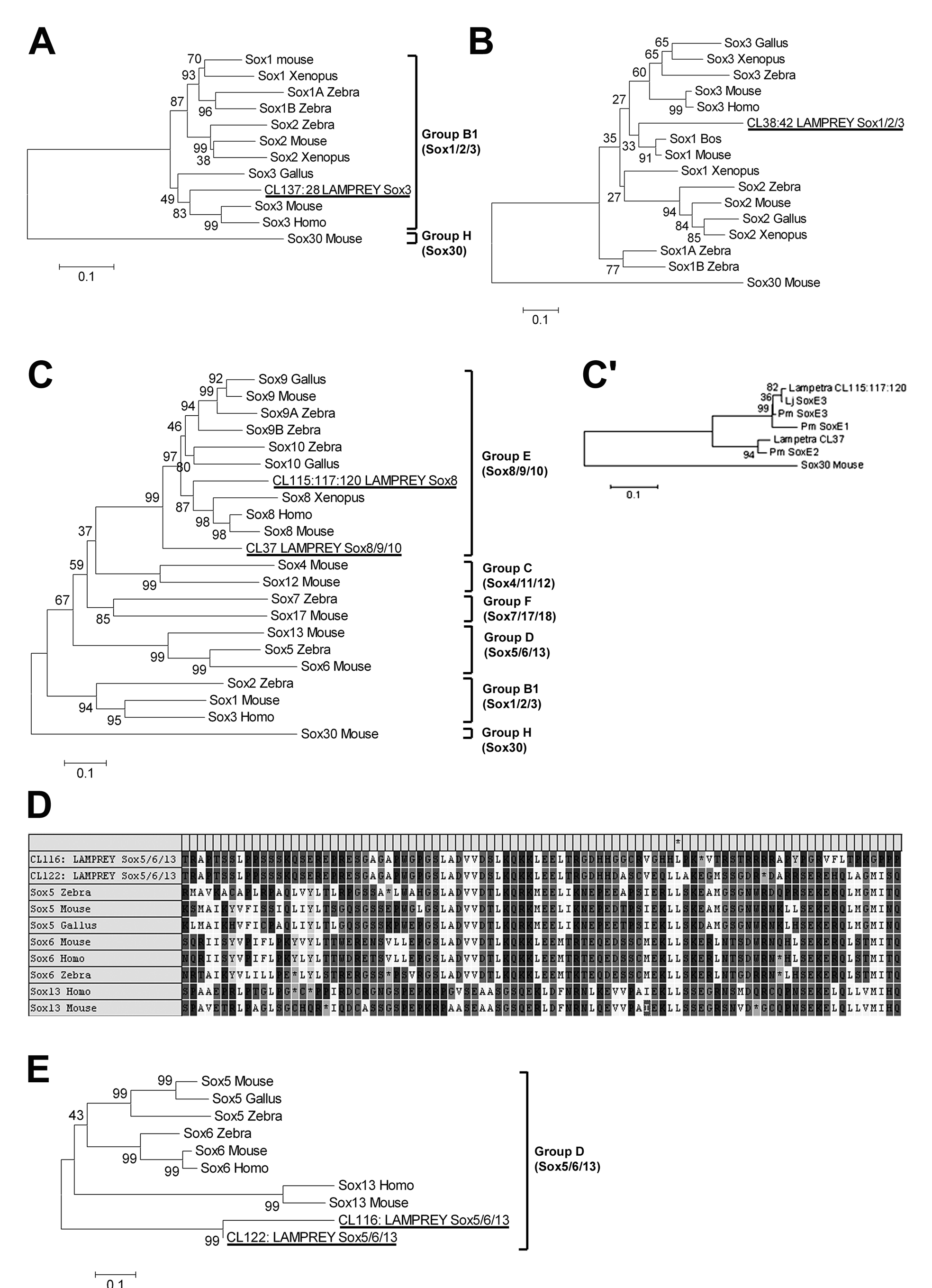

Supplement: Figure S5 — Phylogenetic analysis of Sox family member clones. A, clones 28 = 137, shown in Figure 2LL', correspond to a lamprey Sox3. B, clones 38 = 42, shown in Figure 2LMN, correspond to a clear SoxB1 (Sox1/2/3) lamprey factor, but with poorly supported orthology relationship. C, C', clones 115 = 117 = 120 and clone 37, shown in Figure 5A–D, correspond to two lamprey SoxE (Sox8/9/10) members. The former is Sox8 and the latter has again a non-resolved orthology relationship. C' shows a tree including only lamprey SoxE group members, and allows to propose orthologies with previously isolated Japanese lamprey and Petromyzon SoxE2 and SoxE3. D and E, clones 116 and 122, shown in Figure 5E–H, are two lamprey group D (Sox5/6/13) members. They share a 79 amino-acid long identical stretch of sequence, which corresponds to their dimerisation domain, a functional domain which is unique to the SoxD group. Clustal alignment with other vertebrate SoxD members at this level is show in D. The NJ tree in E shows that the two lamprey paralogs emerge at the root of the tree, and may correspond to a lamprey-specific duplication. (0.94 MB TIF) [file pone.0005374.s005.tif]

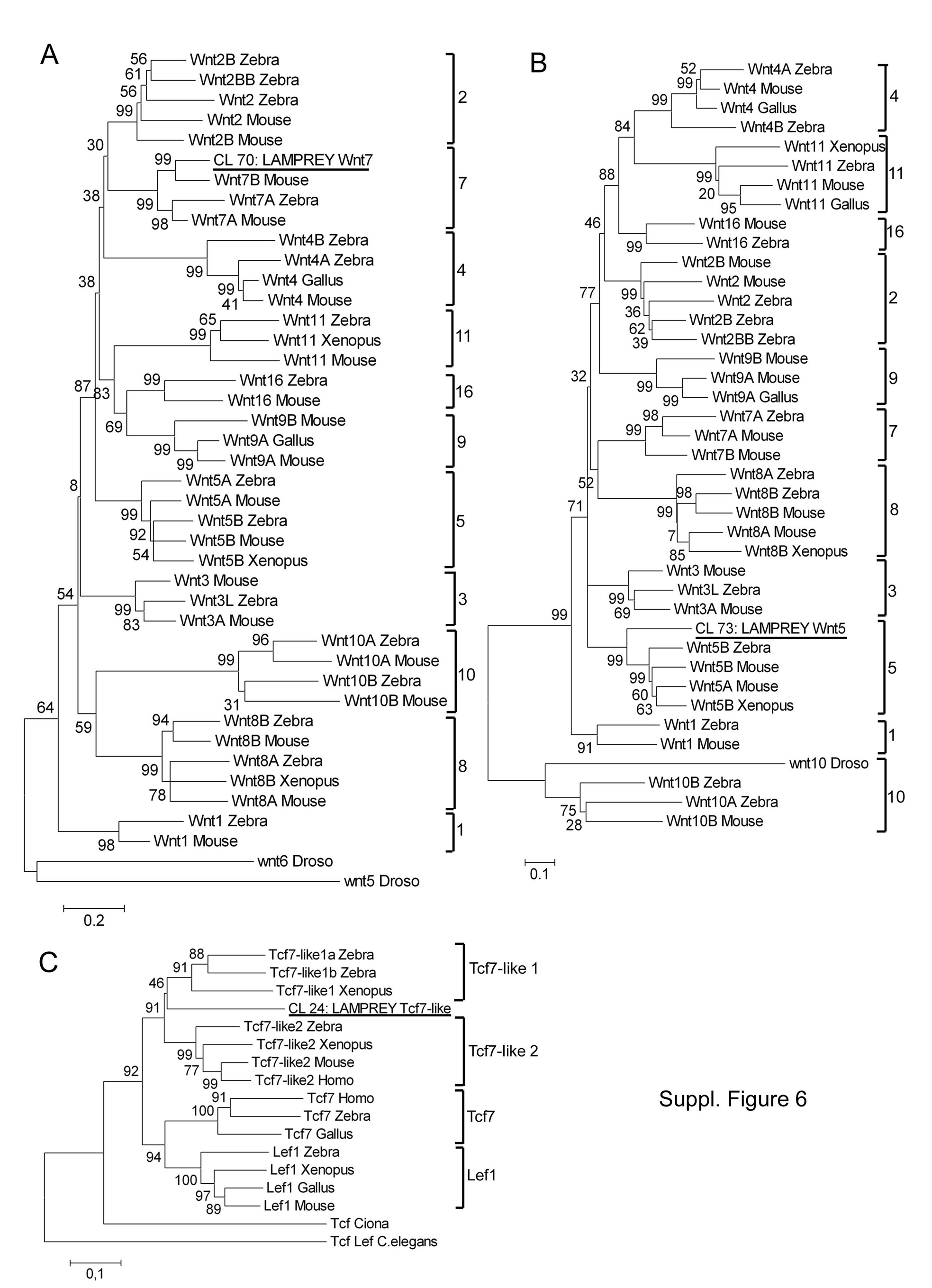

Supplement: Figure S6 — phylogenetic analysis of Wnt pathway clones. A, clones 70 = 1 = 2 (clone 70 is shown in Figure 7ABC) are lamprey Wnt7. B, clone 73, shown in Figure 7DE, corresponds to lamprey Wnt5. C, clone 24, shown in Figure 7FGH is a lamprey Tcf7-like whose orthology is not robustly supported between the Tcf7-like1 and Tcf7-like2 groups. (0.84 MB TIF) [file pone.0005374.s006.tif]

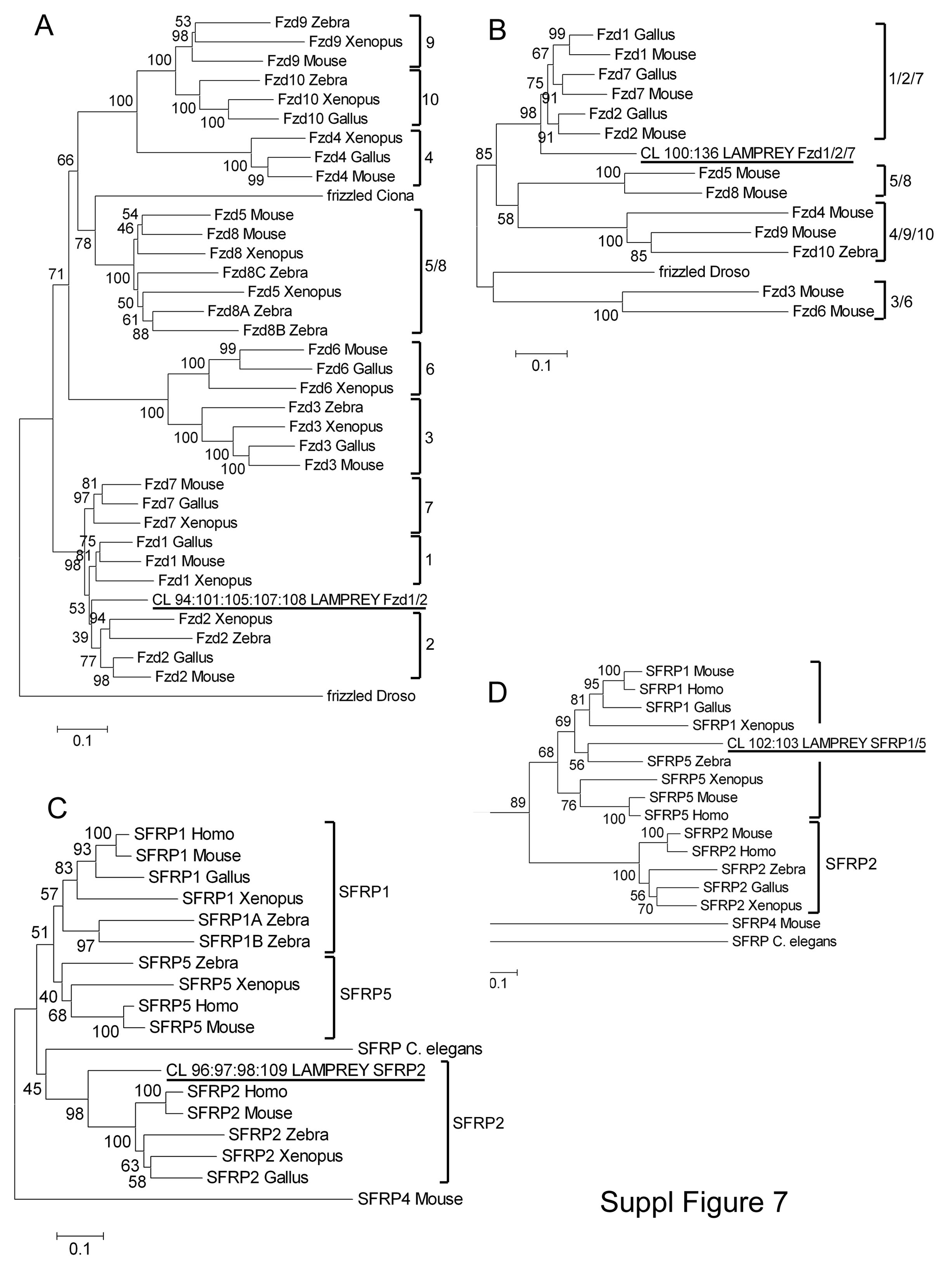

Supplement: Figure S7 — Phylogenetic analysis of Wnt pathway clones, continued. A, clones 94 = 101 = 105 = 107 = 108 (clone 101 is shown in Figure 7I and clone 94 is shown in Figure 7J) are lamprey Frizzled 1/2. Note the organization of the Fzd superfamily, with 4 large subgroups, namely groups Fzd1/2/7, Fzd 5/8, Fzd 4/9/10, and Fzd 3/6. B, clones 100 = 136 (clone 100 is shown in Figure 7K) are also in group Fzd1/2/7, with no support towards one of the 3 possible gnathostome orthologies. Clone 95 (tree not shown, in situ hybridization presented in Figure 7LMN) is in the same case, and identified as an Fzd2/7 member. As a result of this analysis of Fzd clones, we conclude that there are (at least) 3 distinct lamprey group Fzd1/2/7 members, like in gnathostomes, although their exact orthology relationships are uncertain (see also Table 1). C, clones 96 = 97 = 98 = 109 (96 and 97 are shown in Figure 7RST) correspond to lamprey SFRP2. D, clones102 = 103 (both shown on Figure 7OPQ) correspond to a lamprey SFRP1/5, without robust support toward SFRP1 or SFRP5 orthology. (0.69 MB TIF) [file pone.0005374.s007.tif]

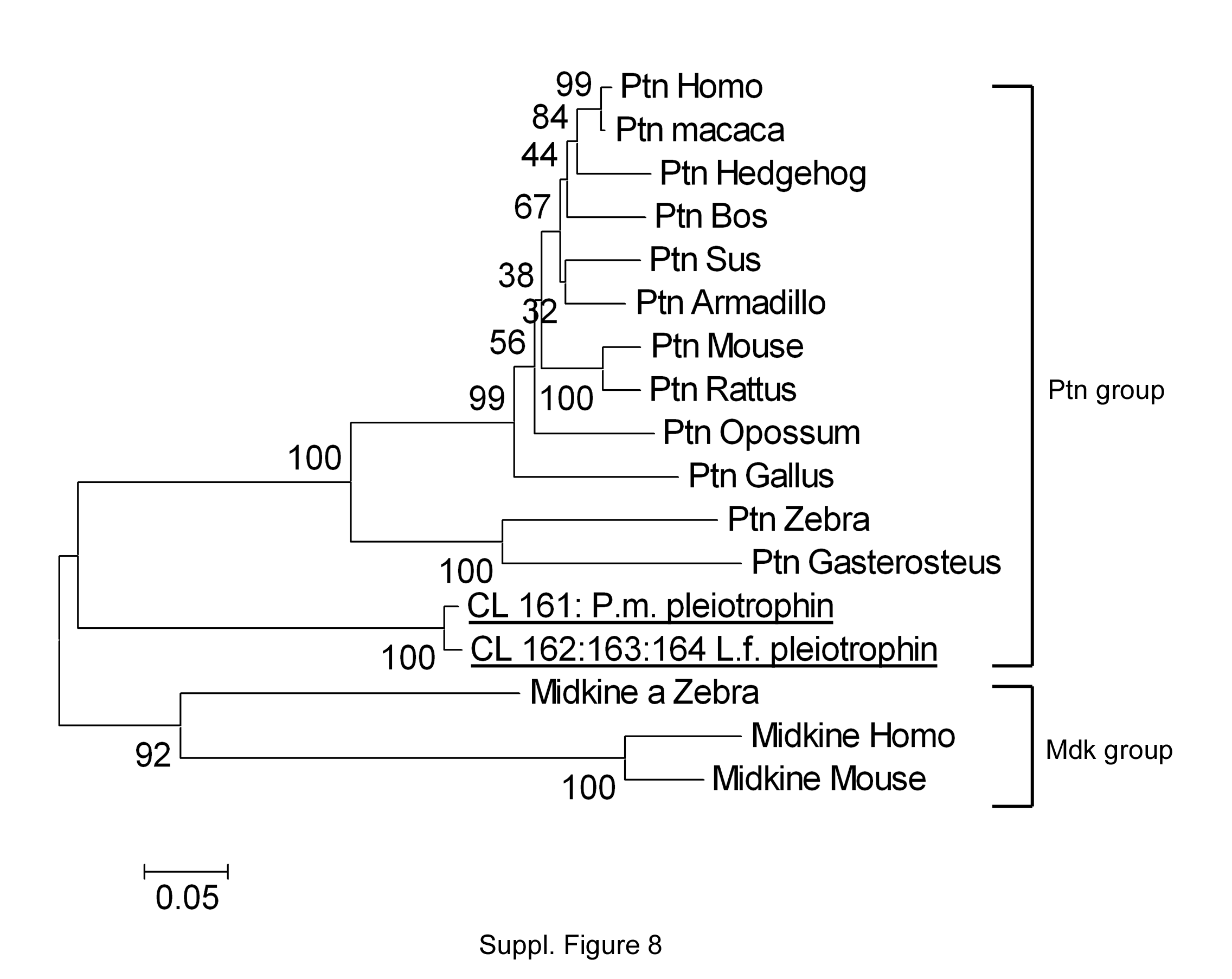

Supplement: Figure S8 — Phylogenetic analysis of pleiotrophin clones. The tree shows that clone 161 (from Petromyzon) and clones 162 = 163 = 164 (from Lampetra) are lamprey midkines and probably belong to the pleiotrophin group. Clones 161 and 163 are shown in Figure 8. (4.13 MB TIF) [file pone.0005374.s008.tif]
